# Kafka, paranoic doubles and the brain: hypnagogic vs. hyper-reflexive models of disrupted self in neuropsychiatric disorders and anomalous conscious states

**DOI:** 10.1186/1747-5341-5-13

**Published:** 2010-08-20

**Authors:** Aaron L Mishara

**Affiliations:** 1 Department of Psychiatry, Clinical Neuroscience Research Unit, Yale University School of Medicine, New Haven, CT 06519, USA

## Abstract

Kafka's writings are frequently interpreted as representing the historical period of modernism in which he was writing. Little attention has been paid, however, to the possibility that his writings may reflect neural mechanisms in the processing of self during hypnagogic (i.e., between waking and sleep) states. Kafka suffered from dream-like, hypnagogic hallucinations during a sleep-deprived state while writing. This paper discusses reasons (phenomenological and neurobiological) why the self projects an imaginary double (autoscopy) in its spontaneous hallucinations and how Kafka's writings help to elucidate the underlying cognitive and neural mechanisms. I further discuss how the proposed mechanisms may be relevant to understanding paranoid delusions in schizophrenia. Literature documents and records cognitive and neural processes of self with an intimacy that may be otherwise unavailable to neuroscience. To elucidate this approach, I contrast it with the apparently popularizing view that the symptoms of schizophrenia result from what has been called an operative (i.e., pre-reflective) hyper-reflexivity. The latter approach claims that pre-reflective self-awareness (diminished in schizophrenia) pervades all conscious experience (however, in a manner that remains unverifiable for both phenomenological and experimental methods). This contribution argues the opposite: the "self" informs our hypnagogic imagery precisely to the extent that we are not self-aware.

## Background: The Natural vs. Human Sciences^i^

Cognitive and clinical neuroscience face very real problems about the nature of the human self, how we define and study "self," and treat individuals when the mind, or brain, becomes so disordered that the experience of self becomes disrupted. "Cognitive neuroscience" contains the terms, "mind" and "brain," respectively. These terms remain imprecise due to a fundamental ambiguity that we are both minds, i.e., *being *a self (so-called first-person experience), and brains or bodies, i.e., *having *a self (third-person perspective). The experienced body (and implicated neural pathways) is comprised by both a motoric-body (proprioceptive body-schema), the "I" (as agent), and perceptual-body (exteroceptive body-image), the social "me," united provisionally and *fragilely *by an interoceptive body (the "mineness" of this relationship). "Mineness" is disrupted in hallucinations of a double or *Doppelgänger*. The verbal descriptors "I," "me," and "mine," however, are only approximations of the underlying neural processes [1-3]. We are generally equipped with common-sense folk-psychological views about self and how we experience other selves, which help us get by in everyday situations. Nevertheless, the self has turned out to be exceptionally difficult to define, operationalize and study in neuroscience and related disciplines. Many researchers in the fields of cognitive science/neuroscience refer to the ability to recognize self in the mirror, or make judgments involving oneself as evidence of self, but this is one-sided. Much of the self-awareness literature confuses mediated self-reference of higher order cognition with *being *a self. It addresses the self as object (having a self), not self as subject (being a self). By overlooking this conceptual distinction, self-reference (representational content about self or self-awareness, self as object) is confused with "being a self" (e.g., Gusnard [4]). The current exclusive focus on self as object ("self-representation," rather than subject of the experience) in neuroscience has its roots in the 19^th ^century division between the natural and human sciences. The 19^th ^century dilemma is reflected in what Levine [5] and numerous philosophers following him, call the "explanatory gap" between neural processes and qualia, i.e., what it is *like *to experience phenomenal states.

**Figure 1:**
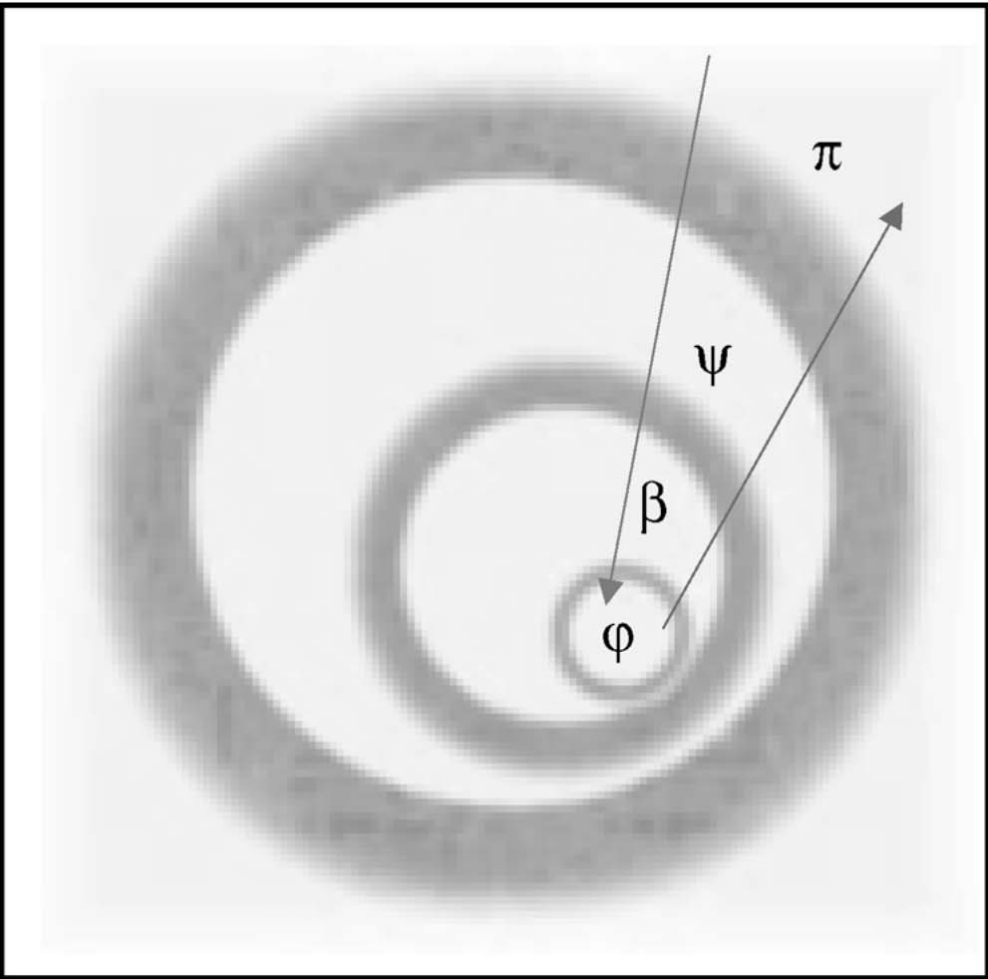
**Methods of the natural and the human-historical sciences**. Opposed directionality between explanation (arrow pointing to smallest circle) and understanding (arrow pointing from smallest circle), indicating the methods of the natural and human-historical sciences, respectively. Natural sciences proceed in terms of the 'classic reductionist hierarchy' from sociology to psychology to biology, chemistry and physics. They generally proceed from larger, rather nebulous wholes to seek out explanatory relationships between ever-smaller parts of these wholes. Conversely, understanding is contextual by situating parts in ever-greater wholes, even if these totalities are ultimately unavailable to the individual perspective but transcend or 'encompass' it. Each discipline requires an 'abstraction, reduction to and idealization (i.e., "naming," Husserl) of the 'objects' or entities of its discipline (which exclude the objects of neighboring disciplines). Gray areas between disciplines indicate interdisciplinary relationships which are often more fuzzy involving destabilizing relationships within interdisciplinary vocabulary and concepts. φ, physis (φύσις), physical-natural sciences; *β*, bios (*βίος*), biological sciences; ψ, psyche (ψυχή), psychological-cognitive sciences; π, polis (πόλις), historical-cultural sciences. From Mishara [6]. Reprinted with permission from Wolters Kluwer.

The '***human sciences' ***(*Geisteswissenschaften*, German translation of J.S. Mill's 'moral sciences') are based on the *'**understanding**' *of the 'meaningful connections' between historical events, whereas the ***natural sciences***, find causal ***explanations*** between postulated natural entities [6]. Figure 1 indicates that natural sciences generally proceed from larger, often nebulous wholes, seeking out explanatory relationships between ever-smaller, strictly defined parts of these wholes. Explanation (e.g. causal/mechanistic, statistical/probabilistic or functional/teleological) tries to establish relationships between subcomponent parts. Conversely, the historical-human sciences generally move 'upwards' from partial views to ever-larger contexts for understanding the matter at hand. Understanding is contextual by situating parts in greater wholes, even if these totalities are not directly available to the individual perspective but transcend or "encompass" it (Jaspers [7]). For example, the historical-human sciences themselves stand in a historical process, which is at the same time the object (as contextual totality) of their study (Gadamer [8]).

Any claim to unify the natural and human sciences is burdened by seemingly insurmountable problems. These include the integration of two opposing directions of method, the effort to make the contextual "understanding" of subjective experience somehow "objective" and testable in the terms of natural scientific explanation, i.e., cognitive and neural processes and mechanisms. However, I make the unconventional claim (requiring justification) that *Kafka's literary writing provides data about the structure of the human self*. That is, it documents processes that are not limited to the individual's experience of self in its historical context, nor the individual's "autobiographical" memory, but reflect the very structure of human self as a transformative process of self-transcendence (in symbolic dream images, a process examined further below) with its own neurobiological underpinnings, i.e., the rudiments for a discipline, "literary neuroscience." *Literature documents and records cognitive and neural processes of self with an intimacy that is otherwise unavailable to neuroscience*.

Such an approach is phenomenological. Founded by the mathematician turned philosopher, Edmund Husserl (1859-1938), phenomenology is the rigorous, methodical description of conscious experience and how the general mental structures derived from its descriptive method may be disrupted in neuropsychiatric disorders and anomalous conscious states. In its step-wise method, phenomenology approaches literary texts as providing "data" about the general structures of consciousness and anomalous states.^ii ^By offering theoretically neutral descriptions of subjective experience (as far as this is humanly possible), it provides a way out of the 19^th ^century dilemma of studying human self as *either *object or subject. Phenomenology proposes rather that the human dilemma is to experience oneself as *both *subject and object [9]. However, its results are provisional and may be refined by more phenomenological investigation or until tested with the experimental methods of neuroscience.

## Why is the Double a Ghost? A Child?

Let us start with Kafka's early story, "*Unhappiness*" (written in 1910) [10]. For our purposes, we may start with nearly any of Kafka's stories to demonstrate ***the same structure of doubling ***[10-14], i.e., of the writer's self in the protagonist,^iii ^and then, a further doubling of the protagonist in the characters he encounters.^iv^

With an approaching November evening, the narrator of "*Unhappiness*" begins by stating that he finds things unbearable (*unerträglich*). He turns away from the window where the street lamps' sudden ***illumination ***startles him. Turning to the ***interior ***of his room, he finds a new goal to pursue in the depths of his own ***mirror ***(*im Grund des Spiegels*). The turning away from the artificial light of the streets to the dark interior of his own room and then his mirror suggest that he is turning inward to examine the "depths" of his own self. The mirror provides no answer to his loneliness and he lets out a scream in order to hear it but no one will answer. (The self-witnessing of expression but its ultimate ineffectiveness in reaching others are major themes further explored below); similarly, reflecting on one's own "inner" self also provides no answer (as in the discussion of *The Bridge *below). The scream meets no resistance to reduce or stop it, even after it has already become silent (suggesting that the scream does nothing to diminish the pain which gives rise to it). As if in response, however, the door opens from the wall (*aus der Wand heraus*) and horses attached to wagons rise (*sich erheben*) in the air. At that moment a small ghost, a child, enters from a completely dark corridor where the lamps are still unlit. She is blinded by the room lit by dusk (*Dämmerung*) and covers her face. The narrator states, "... in short, this visit, though I had expected it, was the one thing needful" ([10], p. 391).

Many of the themes that concern us are announced: the protagonist's loneliness, turning inward, the mirror and scream portending the sudden appearance of a double;^v ^the narrator and his double (i.e., the child-ghost) experience an over-sensitivity to light (photophobia); and the transformed, dreamlike-twilight state (*Dämmerungzustand*). All seem to reflect Kafka's own state as a writer. Curiously, the light of streetlamps and the dusk (which are not particularly intense sources of light) cause discomfort first in the narrator and then in the ghost. We know that Kafka suffered from severe, possibly cluster headaches [15], which may, in part, contribute to his tendency (documented below) to withdraw from excessive stimulation. The suggestion that the narrator's mental state and that of Kafka himself are closely linked is supported by the fact that both live on the third floor [16], p. 88^vi ^The word dusk (*Dämmerung*) refers to the transitional light between day and night when the ghost arrives but it also refers to the narrator's (writer's) state of consciousness, a "twilight-state" (in German, *Dämmerungszustand*), i.e., a transitional state between waking and dreaming or sleep. Notably, the ghost is born in this moment of need, of loneliness, the searching of inner-depths in a mirror, giving rise to *a cry without resonance, or echo, and which never reaches an audience*. The fact that this unusual visit was somehow "expected" by the narrator gives it a dream-like quality. Even though the narrator reports bizarre, unusual events (the ghost, the elevated horses), he does so with the same matter-of-factness as recounting ordinary experiences during waking consciousness. As a result, these unusual turns of events are presented as expected or, at least, not surprising. In this respect, Kafka's narrative resembles a dream.

Dreaming has been characterized as "single-minded" [17]. In waking consciousness, we usually are able to reflect on, compare, or recall experiences, or thoughts, apart from the current one we are experiencing. It is not that these processes are completely excluded during dreaming - a counter-example is lucid-dreaming. It is rather that they are massively attenuated so that dreaming is "isolated" from other capacities or functions of consciousness. One finds a similar inability to transcend one's current perspective, to reflect on, monitor or consider alternative views in acute psychosis of schizophrenia. As in dreaming, one is trapped in the "now" [2,18,19]. Kafka's stories and novels often depict this sort of single mindedness which we find in dreaming. For example, the sudden appearance of the unexpected or bizarre is met with the protagonist's blasé acceptance as matter of course. In his novel, *The Castle*, Kafka describes his protagonist, K., as taking the unexpected as matter of course: "The particular instance surprised K., but on the whole he had really expected it" [20], p. 5.

Once the ghost enters, the (lonely) narrative transitions into a dialogue between narrator and ghost. As in many Kafka stories, the protagonist and the figure(s) he encounters are inextricably related, as if different parts of the same person. Although the narrator states that the visit was expected, he questions the ghost whether she is really looking for him as so many people live in the building. However, the ghost's responses make clear that their relationship is beyond the ordinary. She states, "no stranger could come nearer to you than I am already by nature" ([10], p. 393) (thus suggesting her intimate relation to him as hallucinated double). The narrator then protests, "Your nature is mine and if I feel friendly to you by nature, then you musn't be anything else" ([10], p. 393). That is, he insists that his double mirror him and not the converse.^vi^

Following these self-assertions, the narrator goes to a night-table and *lights a candle*. Since no further dialogue follows, the narrator presumably *releases *himself from the apparition: Through lighting the candle? The verbal self-assertion? Their combination? Leaving the apartment, he meets a neighbor on the stairs and tells him about the ghost. He says to the neighbor (insightfully) that his real fear does not concern the ghost but "what caused the apparition" ([10], p. 394). The narrator continues, "The ghosts seem to be more dubious about their existence than we are, and no wonder, considering how frail they are" [10] p. 395. At this point, the neighbor rejoins that he has heard that one may "fatten up" (*auffüttern*) one's ghost (to which the narrator assents) but then adds salaciously, "Why not? If it were a feminine ghost, for instance?"^vii^

Still we may ask, why is the double a child? A ghost?

## How does the "Brain" produce Hallucinations of a *Doppelgänger?*

Let us compare this story with an actual clinical case of seeing a double (what is termed autoscopy^viii^):

After visiting the grave of her recently deceased husband, a 56-year-old, retired schoolteacher returns home. Upon opening the door, she senses that someone else is in the house in which she is the only occupant. In the twilight-lit room, she sees that another woman is standing in front of her. As she lifts her right hand to turn on the electric light, the figure makes the same movements with her left hand so that their hands meet. She remarks that her own hand feels cold and bloodless from the contact (Mishara [3], paraphrased from Lukianowicz [21]).

As in Kafka's "Unhappiness," the scene is poorly lit and occurs at dusk. It involves a mirroring of the patient's motoric-body^ix ^in that the double anticipates or preempts the subject's intention to switch on the light [2,3,22-25].

Kafka describes looking in the mirror in his diary. As in *Unhappiness*, the lighting is poor. The evening light (*Abendbeleuchtung*) comes from behind and outlines a darkened face which is "...unbelievably energetic, but perhaps only because it was observing me, since I was just observing myself and wanted to frighten myself" ([26], p. 247). The English translation is misleading. The original German states only that the mirror-face "was observing" (not that it "was observing me"), i.e., that the mirror-face was observing Kafka observing himself.^x ^The mirror-image takes on an independence from Kafka. As now a third perspective, *it *observes Kafka's relationship to himself, i.e., a further doubling which "observes" Kafka's own split relationship with himself (see also [27,28]).^xi^

## Can Words Surmount the Rift between One's Own and Others' Experience of Body?

In Kafka's *Metamorphosis *(1912), Gregor Samsa (suggesting, as we have already indicated, Kafka's own name), finds himself - after awakening one night from uneasy *dreams *- transformed into a verminous, gigantic insect.^xii ^While his terrifying form (*Schreckgestalt*) clearly repels others, it becomes useful for Gregor [10], p. 131. It exempts him from the expectations that others impose on him. Having not yet seen him, his family and the firm's procurist are eager to get inside his bedroom with the sole purpose of pressing him to return to his obligations to them. Inside the room, the transformed Gregor speculates on their reaction once they see him: "... he was eager to find out what the others, after all their insistence, would say at the sight of him. If they were horrified then the *responsibility would no longer be his *and he could stay *quiet*. But if they took it calmly, then he had no reason either to be upset, and could really get to the station for the eight o'clock train if he hurried" ([10], p. 98, my emphases). Since the second alternative is implausible, his transformation exempts him from his *responsibility *to support the family.

Aware of its powerfully repellant effects on others, Gregor is alienated from his body image (as he both sees it and how he imagines it to appear to others [3,22-25]). With regard to Kafka's own body image, Gilman [29] asks: "What would Kafka see while looking in the mirror?" (p. 54). By carefully documenting the associations between "illness," "ugliness," and the "feminized" Jewish male body as expressed by the prevailing anti-semitism at the time, Gilman articulates some of the factors which presumably impacted Kafka's own experience of "body image." In Kafka's *The Trial *[30], young girls (including a young precociously sexual, but disfigured child, reminding us of the seductive child ghost in *Unhappiness*) taunt the protagonist, Josef K., through the closed *door *of the painter-artist Tintoretto's studio. The fact that it is an "artist's" studio already gives us some impetus to think that there must be some association between this "artistic" activity and Kafka's own writing activity. The children cry out that Josef K. is too "ugly" to paint [30], p. 150, suggestive of Kafka's own discomfort with body image. Elements of *Unhappiness *are repeated in this scene from *The Trial *(e.g., the door, the child, sexual undertones).

Gregor's sense of agency or motoric-body (i.e., body-schema and its connection with the vital-interoceptive body [3,22-25]) is also compromised: 1) His "numerous little legs... never stopped waving in all directions... which he could not control in the least" [10], p. 92; 2) "...weakness arising from extreme hunger, made it impossible to move" ([10], p. 132). In a manner which at least superficially resembles the muscular paralysis (atonia) of REM sleep dreaming and the hallucinatory nightmare experiences of narcoleptic sleep paralysis, Kafka also describes a similarly incapacitated motoric-body in *A Country Doctor *shortly after the doctor awakens (see below). The transformed Gregor also has sensitivity to light which he avoids (not unlike Kafka himself and the protagonist and his ghostly double in *Unhappiness*).

Gregor stops eating and sleeping to hasten the encroaching death of his hateful-body.^xiii ^In the end, he is just a "thing" (*Zeug) *disposed of by the elderly charwoman. Gregor's father prevents the charwoman from explaining how she disposed of Gregor and the rest of the family appears indifferent.^xiv ^For the others around him, Gregor becomes an object, a body stripped of human subjectivity.

As already emphasized, the claustrophobic conflation of the narrator's and protagonist's perspectives, or the doubling of the author as protagonist, who, in turn, confronts his own doubles during the course of the narrative, are common features in Kafka's stories.^xv ^The protagonist is the closest double to Kafka himself and, as surrogate, is the primary means by which Kafka presumably reaches the reader. Therefore, we will want to clarify why the protagonist's body as the narrator's double (in its hidden reflexive relationship to the very writing process which creates it) is found to be unsubstantial as in the child ghost or is eventually destroyed leading to the protagonist's death.^xvi ^The *Metamorphosis *begins with Gregor's transformation around Christmas and ends with Gregor's death around Easter. The timing is critical for understanding the story and we will return to its possible meaning.

## Does Loneliness Induce the Social Network in the Brain to Become More Active?

The psychological researchers, Epley et al. [31] have implemented studies which suggest that people who feel lonely or lack social connection tend to attribute human characteristics to non-human objects, e.g., machines, pets, or transcendent "objects (such as God)." They "anthropomorphize" these objects "by inventing humanlike agents in their environment to serve as potential sources of connection" (p. 114). The researchers conclude "those who lack social connection with other humans may try to compensate by creating a sense of human connection with nonhuman agents." When they manipulated the mood of healthy subjects by showing them frightening film-clips (*Silence of the Lambs*), the fearful subjects - compared with control-subjects exposed to a non-fearful film-clip - tended to see faces and fear-related stimuli in ambiguous, neutral drawings.

In a similar vein, individuals isolated for long periods (e.g., mountaineers, explorers, sailors, and castaways) report a variant of the *Doppelgänger *experience, the "feeling of a presence." The "double" is felt (but not seen) to be nearby often at a *precise distance *from the subject [3]. The brain's construction of otherness is activated by emotional states of loneliness and fear (including paranoid states and possibly, psychosis). Loneliness and other forms of social deprivation may induce the social networks in the brain (i.e., those brain networks subserving social cognition) to become more active on their own resulting in *Doppelgänger *experiences or hallucinations, as Kafka's child-ghost, but how?

The reduction of social connection leads to the construction of imaginary other(s). The deprivation of sensory stimulation leads to their hallucination. Depriving healthy individuals of sight via blindfolding for prolonged periods leads to visual hallucinations [32]. If blindfolding is sustained for a day or more, both simple (bright spots of light) and complex (faces, landscapes, ornate objects) hallucinations result.^xvii ^Visual and other hallucinations involve activity-increases in the cortical areas that give rise to them [33]. Therefore, cortical excitability may be related to the auditory and visual hallucinations reported by patients with schizophrenia. This concurs with Hoffman's [34] hypothesis of sensory/social "deafferentation" (i.e., reduction of sensory input) in schizophrenia. Hoffman et al. [35] found that slow-frequency repetitive transcranial magnetic stimulation (rTMS), which decreases the excitability of the underlying cortex, reduced the incidence and severity of treatment-resistant auditory hallucinations (when applied to the temporo-parietal region).

Cortical hyperexcitability would be consistent with the deficits in GABA (the major inhibitory neurotransmitter in the brain) revealed by postmortem studies in schizophrenia-patients (Lewis et al. [36]). GABA levels have been shown to reduce within minutes of light-deprivation [37]. It is interesting that the neurobiological evidence supports what phenomenological and existential thinkers e.g., [38,39], have been proposing for years: to withdraw into the self, to remove oneself from others, is paradoxically to discover the other, or others, within oneself, that is, self and other(s) are inextricably related.^xviii^

## What relevance do the neurobiological studies have to Kafka's writings?

Kafka deliberately scheduled his writing during the night in a sleep-deprived state^. ^It is also known that he drew from hypnagogic imagery in his stories [40]. In his *Diaries*, Kafka describes his nocturnal writing as conducted "entirely in darkness, deep in his workshop" [26], p. 518; see also [14]. As Kafka reports, writing without sleep enables access to unusual thoughts and associations which otherwise would be inaccessible: "How easily everything can be said as if a great fire had been prepared for all these things in which the strangest thoughts emerge and again disappear" [26], pp. 293-4, my translation). With regard to this transformed state of consciousness, he writes, "all I possess are certain powers which, at a depth almost inaccessible at normal conditions, shape themselves into literature..." [41], p. 270." Similarly, Kafka writes in his Diaries, "Again it was the power of my dreams, shining forth into wakefulness even before I fall asleep, which did not let me sleep... I feel shaken to the core of my being and can get out of myself whatever I desire. It is a matter of ... mysterious powers..." (cited by Corngold, [42], p. 23). Sleep deprivation may serve as a non-drug "psychotomimetic" model (i.e., producing a psychotic like state in healthy individuals) with attendant changes in dopamine in the striatum and NMDA and AMPA ionotropic glutamate receptor function in pre-frontal cortex [43]. Indirectly, this suggests a possible relationship between intrusive hypnagogic imagery (which is increased with sleep deprivation) and the experiences of beginning psychosis [44], and below.

Kafka's "great fire" suggests a creative process which provides its own illumination even in darkness. It also suggests a state of cortical excitability (and resulting hypnagogic hallucinations) following Kafka's withdrawal from sensory/social stimuli coupled with sleep deprivation. Kafka longs for "complete stillness" (as Gregor in *The Metamorphosis*) eager to separate himself, while writing, from his argumentative family with whom he lived for a good part of his life.^xix ^The Hunger Artist "withdraws deep within himself paying no attention to anyone or anything" [10], p. 268. Kafka is avoidant of unnecessary stimulation, which may also be prompted by his severe headaches [15], and sleeplessness [12], p. 231. However, the withdrawal from photic and social stimulation is also prerequisite for *the self-induction of hypnagogic-like trances*.

Kafka marveled at the automaticity of his own writing. In a letter to his future betrothed, Felice Bauer - whom he persistently tries to discourage, as evidenced by this letter, from wanting to marry him - Kafka writes: "I have often thought that the best mode of life for me would be to sit in the innermost room of a spacious locked cellar with my writing things and a lamp. Food would be brought and always put down ... outside the cellar's outermost door. ... And how I would write! From the depths I would drag it up! Without effort! For extreme concentration knows no effort" [41], p. 156). Here we find solitude, the reduction of sensory stimulation in the cell's darkness, and the automaticity (effortlessness) of the writing process. According to Kafka's own reports, he experienced writing (at least in its initial phases) as automatic, effortless and informed by hypnagogic imagery.^xxx ^When writing is effortless, it is the product of a *trance-state *called "flow" shown to facilitate optimal mental functioning (Csikszentmihalyi, [45]). Kafka writes, "All I possess are certain powers which, at a depth inaccessible under normal conditions, shape themselves into literature..." [41], p. 270). In a letter to Max Brod, Kafka [46] writes that it is "not alertness but self-oblivion [that] is the precondition of writing" (p. 385).

While Kafka was writing, the psychoanalyst, Herbert Silberer, in 1909, conducted introspective experiments [47]. Sleepy one afternoon, he struggles to think through a philosophical problem. To his astonishment, the dream-images which appear while dosing off represent the concepts he was just considering but now in pictorial-visual form (as if in a rebus puzzle). Such images or hallucinations, which are experienced between waking and sleep, are called hypnagogic (hypnagogic from Gk. *hupnos 'sleep' *+ *agōgos 'leading' *(from *agein 'to lead'*, thus a leading into sleep). Encouraged by this observation, he conducts introspective experiments observing what happens while attempting to maintain cognitive effort as best he can while falling asleep. He concludes that the hallucination "... puts forth 'automatically' ... an adequate symbol of what is thought (or felt) at a given instant" p. 196 [47]. Silberer gives the example of falling asleep while thinking through a solution he later admits "forces a problem into a preconceived scheme." His thinking is followed by the hypnagogic-symbolic image: "I am pressing a Jack-in-the-Box into the box. But every time I take my hand away it bounces out gaily on its spiral spring" p. 204 [47]. He interprets the hypnagogic-image to be "autosymbolic." Its content refers to the thought process, mental-function or feeling in conscious awareness that just preceded it before falling asleep. It occurs in the "transitional," "twilight" state between sleep and waking in which hypnagogic/hypnopompic images are spontaneously produced. *Critically, the autosymbolic hallucination requires that the subject is unaware at the time that his own mind is producing it or its symbolic meaning*. Pertinent to our analysis, the phenomenological psychiatrist, Klaus Conrad drew similar conclusions *both from introspective observations of hypnagogic imagery and his clinical observations of paranoid psychosis in early schizophrenia*, see [48,49] and below. For discussion of the limited reception of Silberer's work, see [50].

## Why death? The unfinished inner journey, caught between worlds

Kafka associated the process of writing with the trancelike, effortless hypnagogic hallucinatory process, which produces doubles. *The doubles reflect Kafka, his own writing activity at the moment*, *and presumably result from the increased cortical excitability of a social network *(activated during states of deprivation, sensory, social and sleep). In another letter to Felice, Kafka, however, introduces the relationship between his writing and death: "What I need for my writing is seclusion, not 'like a hermit', that would not be enough, but like the dead. Writing, in this sense, is a sleep greater than death, and just as one would not and could not tear the dead from their graves, so I must not and cannot be torn from my desk at night" [41], p. 279).

In his story fragment which begins with the line, "I was a visitor among the dead," Kafka [51] writes, "It was a large clean vault... two coffins were open, inside they looked like rumpled beds from which people had just gotten up. A desk stood to one side, so that I did not notice it at once; a man of *powerful build *(*mit mächtigem Körper) *sat at it. In his right hand, he held a pen (*eine Feder*), *as though he had just been writing *and had only just stopped. His left hand was toying with a shining watch chain on his waistcoat and his head was bent low towards it (*der Kopf war tief zu ihr hinabgeneigt*). A charwoman was sweeping up the place, but there was nothing to be swept up" [51]. The charwoman instructs the visitor to speak with the man with tilted head but to no avail. The man does not respond but remains *motionless*. Here several elements come together: writing (symbolized by the writing desk and pen) is associated with the underworld (i.e., the depths of the self [1]), but also the narrator's powerful double (i.e., who, unlike the child ghost, does not require "fattening," but whose unavailability, at the same time, disempowers the protagonist), who had just been *writing *is now motionless, with head bent down, suggesting sleep or - in the current vault environment, and the no longer heeded pocket watch - death.

The Prince, in Kafka's story, *The Warden of the Tomb*, is owner of the castle's Friedrichspark and its tomb. He expresses the following desire: "For my family this tomb represents the frontier between the Human and the Other (*die Grenze zwischen dem Menschlichen und dem Anderen*) and its on this frontier that I wish to post a guard" [10], p. 207 (my insert). The prince, however, is surprised to hear that his own warden, elderly and *exhausted*, already guards the tomb. Moreover, the old man reports that he neither dreams nor sleeps *but must remain awake each night *(as Kafka himself in his writing). He must hold "wrestling bouts" with the *ghosts *(i.e., doubles) to prevent them from escaping from the park. As we shall see in Kafka's "Burrow," there is a nebulous boundary between worlds (and states of mind) which must be protected, overseen, fortified, in what the Prince's chamberlain calls, "a real guarding of unreal things beyond the human sphere" [10], p. 207. In his stories *Unhappiness *and *The Warden of the Tomb*, Kafka's writing gives rise to ghostly doubles. This process may reflect the excitation of brain areas responsible for social experience of others and possibly, hypnagogic hallucinations. But why does Kafka make the further move of connecting his writing with death?

In *A Country Doctor*, which unfolds like a dream, the doctor is prepared for a *journey *(*reisefertig*) to treat a seriously-ill patient some ten miles away. His horse, however, has died *during the night *due to *overexertion *(perhaps reflecting Kafka's own nocturnal exertion). As he stands in the courtyard, he finds himself more and more *unable to move *(*immer unbeweglicher werdend*) and the snow starts to cover him. The previous irreversible changes during the night and current immobility (both recalling *The Metamorphosis*) describe Kafka's own being transfixed by the hypnagogic imagery he records (but also shapes) while writing. The doctor then kicks at the rotting, broken-door of his pigsty unused for years in an absent-minded (*zerstreut*) manner (perhaps again reflecting Kafka's own trance-state). From these small quarters, a man, crouching, emerges, *crawls out on all fours*, suggesting infancy or *a self-generated birth*. He asks whether he should hitch the horses, which soon follow from out of this same small "door whole" (*Türloch*) as if emerging from a birth canal in a strange process of twin birth to which we will return when discussing *The Burrow*. The man, a servant, who eventually overpowers the doctor, is often interpreted as representing the doctor's/Kafka's primitive instinctual side. *He is somehow acquainted with the doctor's thoughts and wishes without the doctor having to express them *[14], p. 120. In a dream (or fictive narrative) all characters are to a certain extent projections of the dreamer's/author's mind. Therefore, the figures as expressions of the same self, should in principle, have access to the thoughts of any other character. We see a similar feature in *both *paranoid delusions and autoscopic-hallucinations in which others (e.g., the autoscopic-doubles, as intimately and inextricably linked with self) have access to one's own thoughts/intentions, even anticipating them [3,48].

If the servant at first helps the doctor, he becomes impertinent, and bites the face of the servant-girl. The doctor warns: "You're coming with me... or I won't go, urgent as my journey is" [10], p. 221). In response, the servant claps his hands and the doctor's carriage is carried away by the horses like a piece of wood in a strong current (*wie Holz in die Strömung*). The doctor's ineffectiveness and ultimately passive role, which only increases as the story develops, is not unlike a dreamer who suffers the automaticity of the dream's events.

The doctor then encounters a second double quite different than the servant, a young male patient, who, without shirt, places his arms around the doctor's neck and addresses the doctor as "*Du*." These are unusually familiar gestures for a first meeting.^xx ^The ensuing dialogue occurs like a "conversation with oneself" (*Selbstgespraech*) [14], p. 125. The youth asks the doctor to let him die, but the doctor believes there is nothing wrong with him. However, once the doctor sees the fatal seriousness of the wound^, ^the youth, "quite blinded by the life within his wound," reverses his request: "Will you save me?" [10], p. 224). That is, precisely in their being linked, they reverse their positions with regard to one another, a reverse mirroring, in which each takes the counter-position to his complement. The wound is described as pink (*rosa*), and we are meant to think of the doctor's servant girl whose name is Rosa. When the doctor first sees the wound, he is tempted to whistle softly (*Wer kann das ansehen ohne leise zu pfeifen?*) Kurz [14] writes, "the worms turning in the wound and the whistling are both expressions of erotic desire and death" (p. 127). However, there is also "life within his wound" [10], p. 224. Therefore, it not only suggests death, but also procreation and ultimately, *birth*.

**Figure 2:**
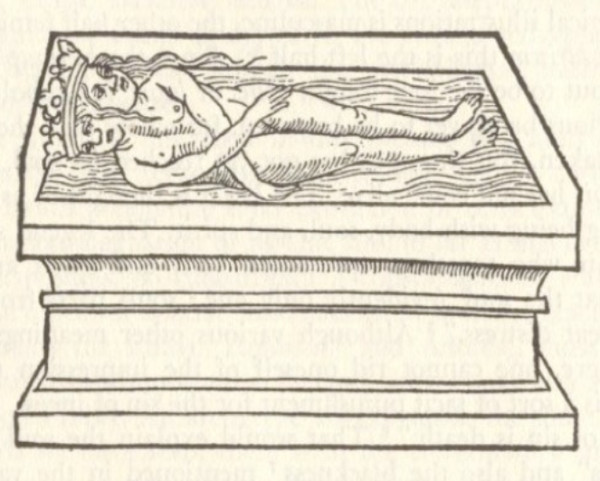
**Woodcut from the Alchemical Work, *Rosarium Philosophorum *(1550) indicating one of the stages in the alchemical process: the Tomb**. This resembles the strange union between the doctor and his patient in Kafka's *A Country Doctor*, which is at once a dying and being reborn.

The family and village-elders strip the doctor of his clothes and place him next to the boy on the side of his wound. Their bed also has the meaning of a coffin. We have seen Kafka's association of bed with coffin in his fragment, "*I was a visitor among the dead*" [51]. In Jung's [52] analysis of the woodcuts in the alchemical work, *Rosarium Philosophorum *(1550), one of the stages in the alchemical process is depicted as two figures, King and Queen, who are joined into one figure in a tomb in a similar manner to the strange union between the doctor and his patient in *A Country Doctor *(see figure 2).

**Figure 3:**
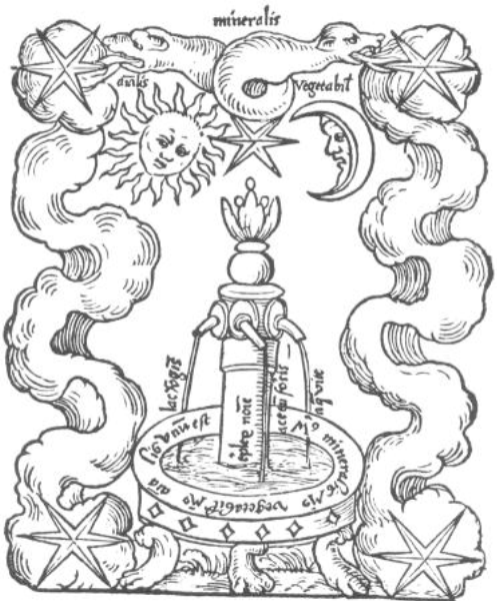
**Woodcut from the Alchemical Work, *Rosarium Philosophorum *(1550): the Mercurial fountain, which Jung (1969) interprets as symbolizing the self as the ongoing integrational effort of a conjunction of opposites**.

With regard to another woodcut, Jung [52] describes the symbol of the mercurial fountain in the alchemical text as a symbol of rebirth or transformation of the self (see figure 3): "the gushing up and flowing back of the Mercurial fountain (an alchemical symbol) within its basin completes a circle, and this is the essential characteristic of Mercurius because he is also that serpent that fertilizes, kills, devours itself and brings itself to birth again" [52] p. 48 (my parenthetical insertion). This "[w]holeness is a combination of I and You, and these show themselves to be part of a transcendent unity, whose nature can only be grasped symbolically as the symbols of... the coniunctio *Solis et Lunae*" i.e., the conjunction of sun and moon, male and female, conscious and unconscious. However, "[t]hese images are naturally only anticipations of a wholeness which is, in principle, just beyond reach." [52], p. 157. Jung's observations about the symbolism of self parallels - in what Jung himself calls "syncretism" - the work of his contemporary phenomenological psychiatrists and neurologists. As I will document, Jung's concept of self as rebirth overlaps with the phenomenologic-existential view of self as ongoing self-transcendence or self-displacement of one's current position. Jung's contemporary, Viktor von Weizsäcker, neurologist, phenomenologist, sense-physiologist, and celebrated "founder" of psychosomatic medicine in Germany, writes that we only come to awareness of self, not as a pre-reflective given, but reflectively. We require and obtain self-awareness only when its presumed pre-reflective unity is threatened, and we only 'resolve' the crisis by paradoxically letting ourselves go: "We only first really notice our own subjectivity when it is threatened to dissolve in crisis. ... The subject is not a firm possession but must be acquired anew at each moment to 'possess' it. ...[T]he *unity *of the subject is only first constituted in its ongoing incessant reestablishing itself in crisis and its own infirmity" [53], p.173 (my translation).

*The self, which comes to expression in doubles through the spontaneous production of symbolic hypnagogic, or dream images, is at once a self-transcendent and a self-regenerating process*. The sharing of the bed with the youth, the symbolism of self-generation of the wound, the youth's reversal of attitude from wanting to die to wanting to live (in contra-step to the country doctor's impressions about the severity of the wound) all suggest the rebirth of the self (as self-transcending, self-regenerating). It is a journey which neither the doctor, (nor any of Kafka's characters, who find themselves - precisely as *fictional *stand-ins for Kafka himself - on unending journeys, (e.g., *The Hunter Graccus*, *The Bucket Rider*, see below) are able to complete.

## Hypnagogic Labyrinth: Conduit for Rebirth?

According to Dorothy Diamant, with whom Kafka was then living, *The Burrow *(1922-3) was composed in a single night. As one of his last stories, it is incomplete. Along with other manuscripts, Diamant may have burned the ending at Kafka's request- in front of his eyes- to purge his soul from "ghosts" (Murray [54], p. 372).^xxi ^*The Burrow *may reflect the newly acquired contentment and reduced loneliness through the relationship with Diamant, but also (as Diamant interprets the story) his fears of returning to the old life bereft of this contentment. Kafka writes in the voice of an unidentified animal who constructs the burrow, "I live in peace in the heart of my burrow, and meanwhile from somewhere or other the enemy is boring his way slowly towards me" (cited by Murray [54], p. 371).

In remarkable interweaving, this story contains the various leitmotivs we have discussed up till now: duplication of the author's self in the animal-narrator who builds the burrow, Kafka's writing as work or building reflected in the animal's construction of the borrow, the relation between literary writing and the transformed mental-state during dreaming,^xxii ^the withdrawal from sensory/social stimulation, reflected in the animal's desire for "stillness" and removal from others ^xxiii ^*which eventually reverses into a paranoid-state*,^xxiv ^and a theme, which so far has remained implicit, *the yearning for rebirth of self*.

The architecture of the burrow is complex: 1) the central *Burgplatz *(alternatively translated as "Castle Keep," "Castle Court" or "Castle square"), is located "not quite in the middle." While the rest of the burrow is "the outcome rather of intense intellectual rather than physical labor," the "Castle Keep was fashioned by the most arduous labor of my body in all its parts" [10], pp. 321-8, translation modified;^xxv ^2) smaller chambers for provisions; 3) connecting, crisscrossing passages and 4) an entrance and exit (see Kurz, 2007, p. 341). "What most disturbs the animal 'I' are the entrance and exit to the burrow... secured by a labyrinth" [55], p. 337). All the more remarkable that the animal-narrator should choose - after discovering that the *rustling-noise *of the invading other has penetrated as far as his Castle Keep - to perch himself just below the flimsy moss-covering (which alone keeps out the external world): "I stray so far that I find myself at my own labyrinth... Here under the moss-covering is perhaps the only place in my burrow where I can listen for hours and hear nothing. A complete reversal of things in the burrow; what was once the place of danger has become a place of tranquility..." (p. 352). Kurz [55] comments on the animal narrator's preoccupation with the "rustling-noise" (in German, *rauschen*, also suggesting, the German word, "*Rausch*," intoxication, a "rush" or transformed state): "In silence, we can already hear our own circulation rustling" (p. 344). The psychologist, Jerome Singer [56] observes that hypnagogic hallucinations "may be characterized by a great rustling in the ears which sounds as if there are huge crowds in the street." In *The Burrow*, the animal at first believes that the rustling-noise "...cannot be from a single animal... it must be ... a huge swarm of little creatures..." [10], p. 347. In *The Castle*, the protagonist, K. while trying to make a telephone call to the castle, hears a buzz from the other end "like the hum of countless children's voices-but yet not a hum, the echo rather of voices singing at an infinite distance. . ." [20]. Kafka's description in these passages of acousmatic sound, i.e., sound without identifiable source, has recently captured the interest of music theoreticians (Brian Kane, Department of Music, Yale University, personal communication). As we examine further below in Kafka's story, *The Bridge*, the sound of rushing water figures in a manner that will connect it to the death/rebirth motif in *The Burrow*.

As we have seen in *A Country Doctor*, the doctor, in a moment of desperation and absent-mindedness, *kicks *in the pig-sty door only to discover that the man-servant crawls out, later followed by what seems to be the most peculiar twin birth of two unearthly horses. Kurz [14] observes that "the wriggling out of the horses from out of the door-whole (*Türloch*) describes a process, which has an erotic meaning" (p. 121, my translation). But it is not only that, the process also suggests a birth. As Kafka writes, "the two horses, enormous creatures with powerful flanks, one after the other, their legs tucked close to their bodies... by sheer strength of buttocking squeezed out through the door whole which they filled entirely" [10], p. 220 (in the original German: *nur durch die Kraft der Wendungen ihres Rumpfes aus dem Türloch, das sie restlos ausfüllten*). With their limbs close to their bodies, like fetuses, they fill the whole completely from which they press themselves outwards *in sequence*. In *The Burrow*, the rather fragile moss-covering (*Moosdecke*) which conceals the animal's "whole of safety" (*Rettungsloch*), suggesting the pubic area, has this double meaning of eroticism and birth. But in the manner of originally 'unconscious' symbols with their attendant "overdetermined" meanings (as they emerge in the initially hypnagogic or spontaneously experienced narrative image during trance-like writing states), the pig-sty door and the moss-covering have still *another meaning beyond the rather obvious suggestion of the female birth canal*. In a manner yet to be examined, they indicate a threshold ("door") between worlds and states of mind and thus refer reflexively to the very writing process that gives rise to them.

The burrow's architecture (*der Bau *(German title for the story) = *Körperbau*, bodily frame) suggests human anatomy. The animal as fetus - resting just under the moss-covering (*Moosdecke*) - travels the birth canal in a rebirth process. Once the animal leaves the burrow, he finds himself in the predicament of how to keep watch on it (from outside) and, ***at the same time***, safely return to it. He ponders that he requires a double, "someone of my own kind ... a hermit" (in German, *Waldbruder*, which contains the word "brother," suggesting a twin or double). Even if this double, however, were to keep watch for him, while he returned to his safe-haven, "I think I would refuse to let him in, even though he alone made it possible to get into the burrow... for either I must let him go in first by himself, which is simply unimaginable, or we must both descend at the same time, in which case the advantage I am supposed to derive from him, that of being kept watch over would be lost." Were the double to remain outside, the animal would also be unable to keep watch of him. Thus, entering the whole of safety (*Rettungsloch*) with his double in the burrow must be sequential (a twin-birth!).

Due to his inability to "trust" the hypothetical-double, the animal decides against letting him enter the burrow.^xxvi ^Rather than doubling the self, he envisions doubling the entrances ("*verdoppeln zwei Eingänge"*). After he descended the one, he could rapidly exit the proximate one and thus quickly achieve a perspective on himself and his construction: "I should have so constructed the first passages that it had two entrances at a moderate distance from each other, so that after descending through the one entrance with that slowness that is unavoidable, I might rush at once through the passage to the second entrance, slightly raise the moss covering, which would be so arranged as to make that easy, and from there keep watch on the position..." (p. 338). The disproportional speed with which the animal is able to enter the one whole and escape from the other suggests the difference between the prolonged gestation of birth (entering) and the state of death (exiting) which, once accomplished, is sudden and irreversible. In the story, *A Bridge *(discussed in the next section), as well as here in *The Burrow*, *the attempt to create a double-perspective on oneself through reflection, or (retrospective, successive) self-observation (in time) ultimately fails*.

Why has the animal constructed a labyrinth just beneath the moss-covering? It is a labyrinth which causes the animal ***physical ***pain to traverse: "So I must thread the tormenting complications of this labyrinth physically when I go out, and ...as sometimes happens, I lose myself for a moment in my own maze..." [10]p. 333, translation modified).^xxvii ^The labyrinth motif may refer to Virgil's *Aeneid *(Book VI) (cf. 55, p. 339). Here, Aeneas begins his trip to the underworld by entering through the Cumaen Gates fashioned by Daedelus. The latter had painted on the Gates' surface the labyrinth he constructed in Crete to imprison the Minotaur. Some commentators point out that Virgil's attentive description of Daedelus' labyrinth painting delays Virgil's poem "needlessly." W.F.J. Knight [57], however, counters that it is "an effective poetic symbol, suggesting the difficulty and confusion involved by a journey into the earth" (pp. 21-2). In a noteworthy integration of classical philological scholarship and archaeology, Knight traces the history of the labyrinthine symbol. He finds the first rudimentary instance of the symbol in the spiral shapes carved into stone placed before and inside prehistoric burial caves in which the dead were placed in fetal position. The spirals had an apotropaic function of trapping evil spirits who become caught in their labyrinthine structure while entering the caves. Knight continues to trace the defensive labyrinthine spiral in the ancient city of Troy's battlements (recounted in *The Iliad*), the "rope dance" performed before the city, and the preserved maidenhood of Troy's virgins symbolizing the city's impregnability. Therefore, it seems fitting that Virgil depicts Aeneas - a Trojan general, who escapes the fallen city of Troy and on his way to eventually founding Rome, - as confronted with Daedelus' depiction of the labyrinth *before entering the underworld*. Nevertheless, the labyrinthine spiral has still another meaning. In summarizing the various sources for his argument, Knight concludes that the labyrinth has the meaning of "access to the earth mother," depicting the earth mother's intestines and thus the promise of spiritual and physical rebirth. Ancient "Greek initiation contained the idea of rebirth by entry into the earth, originally by a cave" [57], p. 53).

Similarly, the moss-covering, labyrinthine-intestines just underneath the covering, and the internal architecture of the burrow suggest the female reproductive anatomy and, indirectly, Kafka's own creative writing process as a rebirth of self. This is accomplished in terms of the autosymbolic function of hypnagogic imagery, but is a process, which - for reasons yet to be discussed - remains incomplete.

Once the animal returns to his burrow, he experiences renewed vigor. This is presumably due to the rebirth-process symbolized as a descent into the underworld of the burrow, which must painfully traverse the convolutions of its own labyrinthine structure: "I have changed my place, I have left the upper world and am in my burrow, and I feel its effect at once. It is a new world, endowing me with new powers, and what I felt as fatigue up there is no longer that here" (p. 341).

As far as I know, Kafka's apparent wish for rebirth is not addressed in the secondary literature but appears to inform Kafka's writings *as if the writing itself were a kind of rebirth of self accomplished through its hypnagogic-doubling process*. As we have examined above, Gregor Samsa's metamorphosis begins at Christmas (Christ's birth) and ends with his death around Easter (Christ's resurrection). Kafka's symbolic-images of journey or rebirth indicate a threshold between worlds or mental-states. To experience rebirth through writing, through the spontaneous, symbolic self-transformation of hypnagogic-imagery, requires a different mental-state, a trance-state open to the unconscious, symbolic formation of images as an inner (transformative) "journey" of the self (cf. Jung, [52]).

## Quest for Wholeness: Narrative as Doubling Self

The structure of the self is organized in terms of its relation to others (intersubjectivity). For Husserl [58], intersubjectivity is possible through doubling my experience as "body-subject" (in German word, *Leib*, related to *leben*, to live). "This is so because my body is already always there in the perceptual field as body-subject" [58], p. 62, my translation). My body serves as the prototypical body-subject (*Urleib*) for how I experience others as embodied whereby I attribute to others the same inner relationship to their bodies as I do to mine. ***Intersubjectivity is a doubling process***. As the French phenomenological philosopher, Merleau-Ponty [59] writes: "The other is born from my side." The phenomenological psychiatrist Jaspers [60] reports a schizophrenia patient who experiences this quite literally: "I had the feeling that somebody was inside me and then, how would you say it, left me by my side?... If I stood up, he stood up. If I started to walk, he started to walk. He always remained at the same place [behind me]. If I turned around to see him, *he also turned around at the same time *so that I was unable to see him. ..." (p. 415, my translation). Others are doubles of my embodied self. It is not surprising that loneliness or fear induces the social network in the brain (responsible for my experience of other minds) to actively produce imaginary doubles of the self. The cognitive neural mechanisms underlying the self as a process which spontaneously produces hypnagogic images of doubling have their basis in Husserl's theory of embodied intersubjectivity, an alternative to the prevalent theory of mind construct in cognitive psychology [3,61]).

The fictional narrative (as a hypnagogic-doubling process and underlying neural activity) symbolizes its own incompleteness by not being able to impart the subjective self (as it is experienced on the inside) to an audience. Kafka's story, *An Imperial Message *suggests the hopelessness of the subjective-self ever reaching the audience by means of words, or the narrative-text: Despite the King's messenger's athletic efforts to deliver the message from the *dying *king to you, the reader, "...still he is only making his way through the chambers of the innermost palace; never will he get to the end of them. ... But you sit at your window when evening falls and dream it to yourself" [10], p. 5, my emphases. The writer and his audience have only language to convey subjective-experiences. With its presumably common neurobiological mechanisms, dreaming, transcends the division between minds encapsulated by their respective physical-bodies, but remains - as Binswanger observes - a private universe (*idios kosmos*) [19,62], thus, "you... dream it to yourself" [10]. Kafka's imagery symbolizes the structure of self as a process of self-transcendence which is condemned to remain incomplete or partial. The child-ghost in *Unhappiness *refers to the process of symbolic self-transformation through the hypnagogic image as reborn (a child) but also one which first requires a symbolic death (the ghost).

Previously, I have written that narrative enables healing of trauma by a process of self-transcendence [18]. The subjective self splits into an "I" who narrates the experiences of a "me" who is, in turn, embedded in the unfolding scenes of the narrative. That is, the narrating "I" is both same and different than the "me" it surpasses in each narrative act. One's self - as totality in the ongoing switching but connecting between (unconscious) body schema and (conscious) body image - eludes conscious awareness but also makes the transcendence of the (painful) past possible. As I have indicated elsewhere, the ongoing switching between body-schema (agentic I) and body-image (social me) may be mapped onto underlying neural pathways (i.e, an ongoing shifting between egocentric and allocentric reference frames [2,3,9]).

*Narrative is the ability to frame imaginary time within real time*. By focusing and narrowing the audience's or even the narrator's attention on scenes in imaginary time (i.e., away from the present context of embodied-sensory experiencing), narrative induces a trancelike state. The anthropologist Levi-Straus [63] describes the shaman's practice of placing a tuft of down into his mouth, biting his own tongue and then spitting out the bloody feather as if it were the pathological "foreign body" extracted from the patient. To cure the patient, he places the patient and the surrounding audience into a trance but, like the narrator, he must put himself into the same trance, i.e., somehow believe his own "performance," to be convincing.

Narrative entrancement (i.e., trance-like absorption or 'attentional captivation' in what is currently being portrayed) is common to all the arts, including performance and visual arts where language is not the primary experience. This ability is thought to have emerged pre-linguistically during the period of *homo erectus *by means of what Donald [64] calls "mimetic culture," i.e., *the ability to tell stories *through gesture and dance before language abilities evolved. Whether the details of Donald's account of cognitive evolution turn out to be correct is not critical to the current argument. Here I wish only to emphasize the *human body's ability to double itself in mimetic narrative *as both the current body expressing ***and ***the symbolic (pantomimed) content it refers to (e.g., one's own body crawling like the panther). This occurs in the trance-like context of the narrative and is a very early form of experiencing the embodied self from both internal and external viewpoints, i.e., as doubled [9]. When we speak, gesture, or write, we are simultaneously recipients, witnesses, of our own communicative efforts. We hear our own voice and partially see our bodily gestures. That is, we take an external, doubled-perspective on ourselves to communicate with others [9]. The questions remain: How does our experience of body enable us to double the self symbolically in narrative? Moreover, how is this symbolic doubling process of bodily self exemplified in Kafka's stories which recount hypnagogic autoscopic images of a *Doppelgänger*? To what extent does this process also help us understand disruption of self in neuropsychiatric disorders [3]?

## Kafka, Phenomenology and Reflective Awareness of Self as Double

Through attending lectures by Brentano's^xxviii ^students in Prague (Anton Marty, Christian von Ehrenfels), Kafka was familiar with the phenomenological movement or at least some of its principles. Nevertheless, he was skeptical about any effort to observe and put subjective experience into words: "There is no such thing as observation of the inner world, as there is of the outer world... The inner world can only be experienced, not described" (Kafka [65], p. 72). Kafka [46] writes in a letter, "For words are poor mountain climbers and poor miners. They do not fetch the treasures from the mountain tops nor those from the mountains' depths" (p. 9, my translation). Kafka's frequent use of metaphors of mining or digging into/merging with the earth is both a symbol of searching the "inner depths" of self, only poorly achieved through the use of reflection and words, but also the wish for rebirth through descent into the 'underworld'[1].

Kafka's story *The Bridge *portrays an insurmountable gap between reflective-verbal description and its pre-reflective "stream" of consciousness. The narrator informs us that he is a bridge, spanning an abyss (*Abgrund*). His body, which is "stiff and cold," suggests an inanimate state. It is nevertheless alive in that his fingers and feet clutch, as if with unflinching "bite" (*festgebissen*) into the "crumbling clay" of the two sides spanning the abyss [10], p. 411. Located at impassable *heights*, he stretches over a ***noisy***, icy stream of trout flowing past "***in the depths***." As evening approaches (the time of twilight mental-states, as in *Unhappiness*), the bridge is *confused*. "The ***roar ***of the stream had grown deeper" (as suggested above, in the multimodal hypnagogic state [3,56]). Then, a person walks, jumps on the bridge: "Who was it? A child? A dream? A wayfarer? A suicide? ... And I turned around so as to see him. A bridge turn around?" By turning around, the bridge collapses into the depths, torn to pieces, skewered (*aufgespieß*t) by the rocks of the rushing stream (i.e., the self's own depths). When the bridge turns back to ***reflect ***(from its lofty ***heights***) on the its own ***passing ***stream of consciousness, the self that just was, the corporeal "me" that the reflective "I" attempts to capture in the reflection, is lost and destroyed in the process.

At first this sounds in direct contradiction to Husserl's phenomenology, but there are surprising points of agreement. Husserl attempts to found phenomenology as a reflective science of consciousness on human subjectivity, which has its own latent-functional structures that enable, i.e., make possible, the conscious experience of meaning. *As a result, the portal of all subjective experience and meaning is the present moment*. However, when Husserl attempts to found his philosophy on this "now," he finds himself in considerable difficulty. Every reflection on a now is itself subsequent to what it reflects and, like Kafka's bridge, human subjectivity collapses before the reflection ever reaches it. Husserl agrees that we do not have immediate reflective awareness of the streaming consciousness as it is occurring. Reflection is always subsequent, after the fact. In the story, *A Bridge*, as in *The Burrow*, the achievement of a double-perspective on oneself through reflection or (retrospective) self-observation (in time) ultimately fails [2]. Reflecting on oneself *presupposes *a splitting of the "I" (*Ichspaltung*) into an currently thinking or reflecting "I" and a reflected (already past!) "me" (Husserl, [58]; see below).

In this paper, I examine Husserl's phenomenology of self only to the extent it addresses the problem of the symbolic duplication of self in hypnagogic hallucination (for more extensive review, see [48]). I have indicated that Husserl attempts to found all conscious experience on an abstract now (the living present), which occurs so "rapidly" (so far as an abstraction may be said to occur at all), that it is not directly available to experience or reflection (except precisely as an abstraction).^xxix ^Critically, the *now *of the "living present" (and its *hidden *absolute transcendental "functioning" subjectivity) *can only be accessed by means of metaphoric description because the abstract "now" is itself "ambiguous" *(Held [66], p. 77): the I as subject is *both *"standing" (persisting in the now) and "streaming" (disappearing into a past). Note that the verbal descriptions of consciousness as "standing" and "streaming" are metaphoric. Thus, *the self experiences a 'rebirth' with each new present upsurge *which passes off as retention into the "night of the unconscious," where it suffers a death (a shrouding over of the retention as it disappears into the past as a "horizon of clouding over" (*Vernebelungshorizont*). It may be "reawoken" or brought back to life as memory in the present consciousness but this new memory itself passes off again to its own death in the streaming consciousness (Husserl [67]; Mishara [68]). Husserl asks, how can the living present be both streaming and standing in original (self-transcending) unity? How can it be both "alive" (in the present) and "dead" as a self already past housing mere ghosts or shades in an underworld [67,68], who, presumably, haunt the present self, for example, as (symbolic) doubles in hypnagogic experience?

We find ourselves in the paradox that the source or fundament of all conscious experience is an *abstract *now moment, which is itself non-experienceable. Similarly to W. James' *metaphoric *description of the present as a melting snowflake, it has already passed in our very becoming conscious of it. The self is an emergent but passive-associative developing of itself (*passiv-azzociative Sich-von-Selbst-Entfalten*) in the ongoing self-displacing *as a standing in the streaming nows*. Husserl [69], p. 53. My own subjective *living *is both the loss of self as subject I just was (now a past me, "split" off from the present moment as the self's double) and the emergence of the I anew (a rebirth) in the current moment. *That is, self as a surpassing of itself with each new now moment is a process of rebirth in which I must paradoxically let go of me myself (now a double or past self "sinking" into the horizon of the past or "subterranean underworld of the unconscious" *[67,68]) *in order to become myself anew as the I who persists *(von Weizsäcker [53]).^xxx ^The I persists as self-displacing, "functioning" center of its own living present, as "standing" (embedded, embodied) in its own "streaming" [66-68]. In these descriptions, the use of metaphor (which we have seen to be 'necessary' in such descriptions) itself becomes an instantiation of its own self-transcending (i.e, by making use of its own indirect, and initially unconscious, reflexive self-reference [2,3,47-49,62]. As we will further examine, this is precisely how the hypnagogic hallucination or hypnagogic dream image takes on autosymbolic meaning [47]).

When controlled conscious processing relaxes, metaphoric images of self arise spontaneously (e.g., hypnagogic images, spontaneous metaphors in narratives) [18,47]. These reflect the self in its dual movement in time as advancing towards the future and letting go of the past, what Husserl calls "I move myself" as the core of self-transcendence in time [2,6]. Binswanger [62] observes that self-transcendence (the "I move myself") is the condition for the ability to distance from one's current experience. Moreover, the ability to transcend one's current perspective is compromised in acute psychosis as well as during dreaming and other anomalous (e.g., hallucinatory) conscious states. Schizophrenia patients delusionally refer to themselves in inhuman or "thinglike" terms, e.g., as a "machine," "computer" or "apparatus" whose sole function is to "register" impressions [62]. This concretization of metaphoric doubling of self is nevertheless an implicit way of preserving (minimal) self in its compromised ability to transcend the current experience but, at the same time, *preserves a distance (no matter how minimally) *by enabling the patient to describe metaphorically, and thus transcend the experience by use of metaphor (no matter how concretely interpreted) [2,3,62]. That is, the delusional metaphor of self as a "registering apparatus" spontaneously refers to the underlying process of self-transcendence, but only in an *unconsciously *reflexive manner, which makes such metaphoric-symbolic doubling of self first possible (as in Silberer's "autosymbolism" [47], but see also [2-4,48,49,62]).

We experience our consciousness as an obligatory displacing itself with each new now. Nevertheless, we do *not *have reflective access to this process which is fundamentally self [6]. As Husserl writes, "the streaming is always ahead *(im voraus*)..." Any representation of self is already past (*having a self*), with its own *closure*. Self as process of self-transcendence is reflected in Sartre's [39] famous phrase, "existence precedes essence" where essence - my self as representation or object - (citing Hegel) is "what is already past": "*Wesen ist, was gewesen ist*" [70]. Sartre [69] had identified consciousness with boundless freedom, a nothingness-- a metaphoric "knife-blade"--which is condemned to continually sever itself from what it just was. Consciousness has to cede each image or representation of itself as already surpassed by the next now-perspective (and its initially open prospective vulnerability to a not yet known future [25]) which replaces it. Self as a process of self-transcendence means that I replace each experience of myself as body as object (the allocentric coordinates of a body-image embedded in retrospective scene-based episodic narrative memories) with the prospective openness to the not-yet-known, the body as subject (the egocentric frame of reference of an on line, anticipatory body-schema, see [3,25], and below).

In a similar manner, Husserl's phenomenology of self points to a "doubling" (*Entzweiung*), or self-dividing of time consciousness [48]. The self-dividing necessary for the memory of a past self is a self-displacing totality, which nevertheless remains *hidden *to itself in its connecting of past and present selves. Metaphors in narratives of self (including Husserl's own descriptions of the self as both "standing" and "streaming") themselves become, as we have seen, instantiations of the self's own self-transcending and thus, its inevitable doubling [2,3,18,48]. Because consciousness is experienced as a "field" or "horizonal" experience with its own background, we are not simultaneously aware of the totality of self that we are as it passes with each new now moment in its successive syntheses of transition (*Überganssynthese*) between now-points [66-68]. Nevertheless, we *symbolize *this totality of self indirectly (pars pro toto) and unconsciously in hypnagogic autosymbolism and the hallucination of doubles.

In a recent publication [48], I indicate that Husserl's phenomenology of self involves a doubling, or self-dividing (*Entzweiung*) of time consciousness (as a self-displacing totality). Husserl requires this doubling to account for how we can have a memory of a past self in the present. In Husserl's terms, whenever we remember something, the memory occurs in the present but refers to a past that transcends this present. That is, each past memory is not only itself (its own "self-givenness") but also part (*Mitglied*) of a *larger transcendent totality, the past*. The past continues to "enrich itself" (*sich stetig bereichendes*) or expand with each new experience *up to the present *(Husserl [67], p. 207). As such the past is *both *immanent to present consciousness in making available *individual *memories that occur at the moment, but also a transcendent "realm of Being in itself," a hidden unity, which any particular remembering presupposes and refers to [48,67,68]. *The phenomenological self is both the kinesthetic-noetic orienting to an emergent affective saliency in its own field but also the totality, which transcends the current moment*. That is, it is both the living present and the transcendent past as *one *totality of streaming consciousness.

As we have seen, the *self *as the totality of the streaming consciousness is *both *the present moment *and *the transcendent past. Any present moment passes off *as retention *into this "underworld of the unconscious." ^xxx ^However, we must also appeal to this same unconscious to retrieve any past now in a recollection. The past (as immanent "object") is continuously instituted anew *in the present *(*immer neue Urstiftung in sich vollzieht*), but as precisely that which transcends the present as its *own realm of Being*.^xxxi ^However, it is through this same past that streaming consciousness constitutes itself as "true Being" (*wahres Sein*) (*ibid.) *and to which we owe the "*idea of a true self*" (*die Idee eines wahren Selbst*). "Before any activity of the I," consciousness "objectifes" itself as past, as no longer *I *but a retrospective *me *(Husserl [67], p. 210). *Paradoxically, the self as the streaming totality is transcendent to present consciousness as its past, but also, in a way that remains hidden, is also this present consciousness*.^xxxii^

In conclusion, *the connection between current and past self is only possible through the retention (of the just past present) disappearing, it's being covered over by the empty retention *[67,68], by the *unconscious *relationship between present and past selves as a self-transcending, or self-dividing, a doubling (*Entzweiung*). That is, to be both present self and remembered past self, we remain unconscious of their relationship which remains a *hidden unity *(of transcendence of the past) in the here and now of the currently affective present, or the present episodic, narrative remembering of what is now past [48].^xxxiii^

## The Body doubles as Symbolic Self

The view I have presented here is in direct contradiction to the French, theologically minded phenomenologist, Michel Henry. The latter has served as a source for recent efforts which attempt, erroneously I believe, to base contemporary neuroscientific concepts of subjectivity on Henry's concept of self as pre-reflective self-awareness. Because this approach, as Henry himself, wants to leap over the accumulated methods and insights of the phenomenological tradition, but in a way that claims putative ties with this tradition, I have called this approach neo-phenomenology [6,71].^xxxiv ^Despite more recent efforts by the neophenomenologists to graft Henry's approach onto Husserl and other phenomenologists, Henry's own work stands *outside *the tradition of phenomenology, even counter to it.

Henry [72,6] proposes that pre-reflective self-awareness, what he identifies with passive auto-affection, is the manifestation of self to itself in its own *immediate *feeling of life. For Henry, this is the givenness of the *subject *to *itself *in the radical passivity or immanence of "auto-affection." That is, Henry's concept of self (as subjective body given to itself in radical immanence as life) presupposes in its one-sidedness precisely the opposition between subject and object and thus - despite hand-waving protests to the contrary by his adherents - remains trapped within the 19^th ^century methodologic oppositions outlined in the beginning of this paper. Henry [74] writes: "there is laid bare a first dimension of experience in which what must be understood as the ground of the psyche experiences itself in radical immediacy, before any 'relation to an ob-ject,' prior to the arising of the world and independently of it" (p. 160). Or: "An immanent revelation which is a presence to itself... understood as an original revelation which is accomplished I a sphere of radical immanence [that] exists by itself, without any real context, [and] without the support of any exterior and 'real' Being" [72], p. 41. Similarly, Henry describes a "radical phenomenology, capable of discerning at the very heart of pure appearance and in the phenomenality of the visible a more profound dimension where life attains itself before the advent of the world..." [75], p. 3. That is, bodily subjectivity "knows" itself as life, incarnation, without needing to take the detour of mediation in externality, temporality, embodiment, otherness or intersubjectivity.

Critical for the current argument, Henry [73-75] critiques the Freudian unconscious as being both a product of, but also lying outside the "metaphysics of representation." It is rather the "body" as alive, as manifesting itself to itself in complete self-awareness (and thus, not an unconscious at all). Here, self is given to itself in the immediacy of life feeling itself; there is no *gap *between self and its own autoaffection as the instantiation of being alive at the moment, an interiority immediately intimate (i.e., without distance) with itself. He [74] proposes that the problem of ultimate grounding rests with the structure of appearing itself in that all intentionality refers back to the ecstatic appearing in which it itself unfolds. Rather, the concept of ipseity,^xxxv ^and its neophenomenological, appropriation as passive self-affection (or so-called pre-reflective self-awareness) is ultimately a matter of faith. It is theological: "Through its phenomenological essence-because it is thereby truth, pure manifestation, revelation-the Life of which Christianity speaks differs totally from what biology studies. ....Since the coming-into-itself of life is its coming into the 'experience-of-itself'...it follows that this enjoyment of self, this 'feeling of oneself' is the first form of any conceivable phenomenality.... This identity between experiencing and what is experienced is original essence of Ipseity." (Henry [76], pp. 34, 56). Similarly, Henry writes (which will have more meaning when we consider his theory of the ontology of the body as "absolute subjectivity"): "The powers of my body do not, therefore, give me the being of the world except on the condition of being known in a knowledge where the concept of the world plays no role whatsoever... The body is present to us in the absolute immanence of subjectivity" [73], p. 93-4.

In his phenomenological analysis of the subjective body as immediately given to itself as autoaffection, the French phenomenological thinker Henry [73] observes that the "subjective body" (as it is given to immanence) and its objective "representation" (the "constituted body") in its scientific study as object to be "two bodies." He asks how can these two bodies be in any way connected or experienced as one: "why do we not have a single body, but, so to speak, two bodies, or if you prefer, why does the being of our body split into an originally subjective being and transcendent being...?" [73], p. 115. However, I experience both bodies as "mine," "which causes this being to be given to me twice" (Henry [73], p. 115).^xxxvi^

Henry's answers that it is only because we are able to experience the body as doubled in this way that we are able to have symbolic language or signification. Henry writes, "Solely the development of these views could lead, in our opinion, to a satisfactory theory of symbolism" [73], p. 92. That is, the relation between the lived body as subject and its double is "symbolic." Note that this is a variant of what we have already said with regard to Donald's thesis (described above) that we are only able to have conscious episodic memory at all because the body is able to double itself mimetically in telling stories, even if these narratives were at first non-verbal but only public performance. However, it is precisely at this point, despite this momentary reconciliation, that we must part company with Henry. The unity between sensing and moving is not, as Henry proposes, given as absolute knowledge or complete coincidence in immanent self-awareness (of an exclusively subjective body given directly and immediately to itself) but remains the "*hidden *unity" of the subject in terms of a Gestalt-circle (*Gestaltkrei*s) between perception and movement (von Weizsäcker [53,77]).^xxxvii^

Following von Weizsacker's insights, Merleau-Ponty [78] describes the famous example of the inseparability of the two kinds of bodily experience - body as subject and body as object - in the oft-cited "double sensation" thought experiment. In this "experiment" one of my hands touches the other hand (which, in turn, touches some third object in the world): "My left hand is always on the verge of touching my right hand touching the things, but I never reach coincidence; the coincidence eclipses at the moment of realization, and one of two things always occurs: either my right hand really passes over to the rank of touched, but then its hold on the world is interrupted; or it retains its hold on the world and I do not really touch *it *- my right hand touching, I palpate with my left hand it's outer covering." (Merleau-Ponty [78], pp. 147-8). I may, at will, alternatively inhabit each of the two complementary attitudes. However, the moment I actively touch my right hand with my left hand, my right hand becomes its object (something touched) and loses its active status. The moment my right hand resumes its active role touching the object, I no longer experience its being touched. Despite Henry's claims for an "absolute unity" putatively given in pre-reflective self-awareness (but somehow also accessible to verbalized reflection) between affecting and affected as the "pure" immanence of passive self-affection, we see that the human embodied relationship to self is better characterized as a "hidden unity," a *Gestalt-kreis *as ongoing self-transcendence, in which the active and passive attitudes to my own touching are mutually exclusive.

In a manner which integrates both von Weizsäcker's concept of Gestalt-circle and the neurologic opposition body schema/body image, Merleau-Ponty [78] writes: By means of ''reversibility . . .alone, there is passage from the ''for itself'' to the ''for the Other'' (i.e., from body for self to the body for others). He adds: ''There is not the For Itself and For the Other. They are each the other side of the other.'' (p. 263). ''The body sensed and the body sentient are the obverse and the reverse . . . as two segments of one sole circular course. . . which is but one sole movement in phases.'' (p. 138, my emphasis). Henry proposes an isolated subjectivity immanent to its own body, yet the moment we are conscious of being/having a body, we are already inextricably related to others and a world. *Contra Henry, there can be no self without another as its converse, its other side. Our only path to ourselves is through embodiment, which includes the other in ourselves (for which we have also found neuroscientific evidence (see above)), a Gestalt-circle of self-transcendence as (reversible) being in the world *[62], what Merleau-Ponty [78] calls "flesh."

## Operative hyper-reflexivity and the myth of the Apollonian

Adopting Henry's concepts, proponents of the hyper-reflexivity model of schizophrenia claim that a pre-reflective or operative "hyper-reflexivity" compensates for the "diminishment" of selfness in pre-reflective self-awareness (ipseity, or passive auto-affection). Here, phenomenologic method is putatively implemented to describe the first person perspective as "pre-reflectively self-aware." That is, the first person perspective is *assumed *to be the same as "pre-reflectively self-awareness." Since I discuss this approach and the current debate in detail elsewhere [6,71], I present an abbreviated account only to the extent that it is pertinent to the current discussion.

A major proponent of this approach, Zahavi asserts that, *whenever *I experience anything, there is a concomitant (tacit) self-awareness. Experience is given as experienced in a first-person manner in which prereflective self-awareness need only be co-conscious (*mitbewusst*). The first-person perspective is *always present *because all experiences are infused (at least tacitly) with the quality of "mineness": "*Any experience *that lacks self-awareness is nonconscious." That is, *consciousness of experience is synonymous with mineness or having a first-person perspective*. Because what makes the experience a conscious experience is by definition the mineness of its (implicit) first-person perspective, it exhibits the principle of 'immunity to error through misidentification' (for review, see Mishara [6]).

Given the tenuousness of Henry's position, however, it is puzzling that these thinkers, who identify themselves as phenomenologists, should base their entire approach on concepts, which clearly go beyond what phenomenological method is able to study. We are reminded of Husserl's [69] own admonition: ""One falls so easily into the mistake of being abstract" (p. 350, my translation).

For *reasons which rest in the phenomenology itself*, the concepts of pre-reflective self-awareness, and its attendant "operative hyper-reflectivity," a position that I have labeled neo-phenomenology [6,71],^xxxviii ^are, in principle, NOT accessible in terms of 1) phenomeonlogic reflective method, or 2) scientific experiment. I begin with pre-reflective self-awareness:

1) Phenomenological method is a stepwise method of *reflecting *on experience. ^xxxix ^Since reflection is inevitably *retrospective *to the experience it reflects on, it is not able to access the putative pre-reflective self awareness without knowing whether reflection itself has transformed the experience, or inserted the very "results" it is looking for.^xl ^As a result, arguments for this construct are indirect, abstract and ultimately, vacuous.^xli^

2) The methods of neuroscience are only able to measure subjective processes and experiences in terms of the subject's responses (verbal, non-verbal, neural). These inevitably involve a "delay" in real time between the experience and the response. Even so-called more direct measurements of neural 'responses' (e.g., fMRI, PET, EEG) inevitably involve a temporal-artifactual delay between what occurs in the brain and the preferred method for measuring it. There is no experimental access to this putative construct which supposedly "constrains" the very method which is unable to test it.^xlii ^Therefore, the construct cannot be established in phenomenological reflection nor falsified with scientific method, and thus not able, as its proponents claim, to "constrain" neuroscience. There is the liability when reflecting on one's own experience that one becomes overly attached to one's own constructs. Husserl's phenomenological injunction, "back to the things themselves," should serve as sobering antidote.

With the recent openness in cognitive and clinical neuroscience to studying the so-called "first person perspective," it is understandable that neo-phenomenological proponents would want to rush in quickly with bold assertions about being able access to first person knowledge to fill this need - or rather, what really is and *remains *an explanatory gap (see Mishara [9]). Once maneuvering themselves into a plausible position imparted to the scientific community at large, but in an esoteric language that only few understand, they defend their pseudo-occupancy of this gap. As I contend in more detail elsewhere, their very efforts to do so, however, precipitously resulted in the assertion of a series of vacuous concepts incapable of being verified (e.g., ipseity, operative hyper-reflectivity, psychosis as "solipsism," the ability to "explain schizophrenia" with non-testable constructs, the abstract and artificial separation of a "pure" qualitativeness from its inextricable relation to the quantitative in everyday cognition (see [53], and below), and finally, the ability to study the schizophrenia patient's subjective experience of negative *signs *(*by definition not directly experienced by the patient but only observable to the clinician*) through the *'self-reports' *of Artaud's literary writings, see [71]). It is curious that the neophenomenological adherents have elected to privilege precisely those constructs, which cannot, in principle, be directly studied either by phenomenological or scientific method. As a result, the constructs appear not to be useful in *both *their application to the psychopathology of neuropsychiatric disorders/anomalous conscious states (including hypnagogic hallucinations as discussed in this contribution), *and *their neuroscientific study. To the extent that the concept of "operative hyper-reflexivity," and its counterpart, ipseity (pre-reflective self-awareness as autoaffection in the sense of Henry) cannot be verified by phenomenologic method, nor falsified by neuroscientific experimental method, they have - although it is always possible that my assessment is incorrect - limited usefulness in the diagnosis, treatment and/or research of schizophrenia.

To summarize the argument so far: Neophenomenology claims "phenomenological descriptions, including pre-reflective self-awareness "*must" *act as constraining conditions for any neuroscientific explanation' of psychiatric disorders" (my emphasis, see Mishara [6], for direct quotation). Regardless of whether the claim of constraint is meant in a strong or weak sense, as it appears the neophenomenologists shift their position, now denying their previously strong claims, see [71], this claim of necessity ("must") errs on two counts: 1) Since phenomenological descriptions are only possible through the phenomenologic reflective method, the construct pre-reflective self awareness is not available to this method; 2) the phenomenological method does not yield "final" results which could be taken wholesale by other approaches, especially neuroscience which proceeds by completely different methods.

There is the related problem that our measurement of conscious experience or other cognitive and neural processes occurs, by definition, in the real time of neural events. That is, *the experimental method is only able to access the subject's experience of self in terms of the subject's responses*. There is no way to directly study pre-reflective self-awareness without probing the subject to elicit a response, and *a response requires a delay in real time*, and thus the preclusion of naïve immediacy in Henry's sense. While it is possible to *study pre-reflective self *experimentally [6], e.g., priming studies, or even reflectively, as I propose in the current contribution, it is not possible, in principle, to directly study pre-reflective self-*awareness *as any intervention (phenomenological or experimental) to access such self-awareness through the subject's verbal reports would require that the subject be reflectively aware, in a now shifted attitude, of what he supposedly experienced pre-reflectively.

Neuroimaging studies of so-called default mode network activity, or resting state, are equally helpless in accessing pre-reflective self-awareness as any post-scanning debriefing about what the subject experienced during the "cognitively unconstrained" or "daydreaming" default mode activity, requires the subject to respond *verbally *and *reflectively *to her previous, putatively "pre-reflective" experience. Moreover, the tenuous equation of ipseity with fMRI default mode activity (as proposed by the neophenomenologists) is invalid due to a host of additional methodical problems [71]). So-called front-loading the experimental subject with phenomenologic reflective skills (as implemented by Lutz and colleagues in so-called neurophenomenological experimental studies) also does nothing to alleviate this methodologic problem of only being able to access the putative constructs through the subject's verbal or other responses. That is, the subject is only able to reflective-verbally report that which is accessible to a reflective attitude - thus requiring a shift in experiencing with different neural circuitry from the pre-reflective experience putatively contained in such reports. As the above claims of direct, "absolute" reflective access to pre-reflective self-awareness, such "front-loading" stands in danger of confounding its results with a "verbal overshadowing" effect [71]. We also know that even the slightest change of attitude from non-reflective to reflective as in the simple paradim "refresh" (developed in Marcia Johnson's lab, Yale University) requires the recruiting of entirely new networks of the brain. This indicates that *even the simplest reflection on our experience requires a different cognitive attitude (as Husserl himself repeatedly emphasized) and different neural processes subserving this attitude*.

Conversely, the phenomenology of the internal consciousness of time has a host of opposite methodological problems. It is performed in the subject's reflection. Its results are abstract and provisional to the *type *of phenomenological "reduction" performed and the ongoing refinement from further reductions which may abstractively isolate this or another layer of experiencing. While the phenomenological researcher may report on the abstracted components of subjective time-consciousness, as they have been isolated verbally according to the step-wise method recounted above, the content of these reports cannot itself be *directly *measured in real time by means of the subject's motor, verbal or neural responses. *What neophenomenology apparently overlooks in its effort to fill the explanatory gap is that attempts to link the phenomenology of subjective experience with the real time conditions of conscious experience (accessed experimentally) can only be related in terms of a Gestalt-circle (Gestaltkreis) *(von Weizsäcker [53]). That is, each approach presupposes but also methodologically excludes its complement as two sides of the same coin. Hence, the privileged neo-phenomenological constructs (e.g., pre-reflective self-awareness as ipseity, or "operative hyper-reflexivity") are themselves untestable and thus, nonfalsifiable. Their claim that they are able to directly "constrain," or even contribute to neuroscience in any way at all, without first providing concepts which are capable of being formed into testable hypotheses, is at best naïve. The neophenomenologic ongoing evident refusal to enter into constructive debate betrays the inhering vulnerability of their position by adopting what I have labeled an "emperor new clothes" strategy (See Mishara [3,6,71]).

The neophenomenological position is that a dimension of "mineness" (or a passive auto-affection) pervades all conscious experience by virtue of being conscious (see [6]). It is therefore a *pervasive *quality of consciousness that cannot be directly measured, and thus not dissociable from consciousness itself. Sass repeatedly argues that the "diminishment" of pre-reflective self-awareness or passive self-affection in schizophrenia, i.e., "ipseity" (or selfness) is strictly *qualitative *and *holistic*, and therefore not captured by the usual ways of *quantitatively *diagnosing the signs and symptoms of schizophrenia. As we will examine, the failure to conceptually separate "diminished ipseity" from its "increased hyper-reflexivity" contributes to its vagueness and the barriers to studying it either phenomenologically or neuroscientifically.

To review, Sass proposes that an operative, pre-reflective or automatic "hyper-reflectivity" (which, as I will explain, is both self-contradictory and implausible at least in the terms of the neuroscience) compensates for the "diminishment" of selfness (ipseity) in pre-reflective self-awareness in schizophrenia. Sass bases his use of the term "operational hyper-reflexivity" on an explicit analogy with Merleau-Ponty's concept of "operational intentionality," to mean "tacitly" or automatically functioning. However, it is not clear to what extent hyper-reflexivity may be considered similar enough to the phenomenological concept of "intentionality" to justify the analogy. Sass' idea is that hyper-reflexivity (or exaggerated self-reflection) may itself become *automatic *in schizophrenia leading to the ''pop out'' of irrelevant background stimuli during early psychosis. However, Sass' efforts to find support for his neophenomenological concepts of ipseity and operative hyper-reflexivity in neuroscientific research (e.g., gamma band coherence, Gray's comparator model, perceptual pop out, default mode activity) are based on vague analogies which appear (in my view) not to understand the original neuroscience (see [71]).

Since ipseity is non-dissociable from consciousness, then compensatory hyper-reflexivity must be unconscious, automatic or what the neophenomenologists call "tacit." While the compensation of diminished selfness in prereflective self-awareness by means of an increase in pre-reflective hyper-reflectivity sounds like it is a *quantitative *relationship, Sass and his co-author, Parnas insist that they are describing *holistic *transformations of subjective experience that *cannot be captured quantitatively *and in fact *cannot be really separated from one another*. That is, *ipseity and automatic hyper-reflexivity are really the same*.

These claims of being able to separate quality from quantity in our pre-reflective naïve experiencing in a manner proposed to "constrain" neuroscience - *without being testable according to neuroscience's own methods *- just perpetuate the divide between the human and natural sciences (as outlined in the introduction). Viktor von Weizsäcker [53] the phenomenological clinician and sense-physiologist, regarded as the founder of psychosomatic medicine in Germany, argues that quality and quantity are inextricably interwoven in dialectical relationship (i.e., *Gestaltkreis*) from the very beginnings in our everyday cognition and the earliest levels of neural processing of a perception or a movement (see [71]). That is, on the level of naïve experiencing, every *qualitative *description is at least implicitly a *quantitative *description. Any particular experience of a perceptual, or feeling quality is implicitly also one of degree of intensity, volume, pervasiveness, etc. That is, on this level, although mutually exclusive *as abstractions*, the two terms, quality and quantity, mutually presuppose one another in inextricable interweaving (*Verschraenkung*) according to the revolving door principle of a Gestalt-circle [53]. All this is merely to indicate that the divisive separation of the human sciences (liberal arts, as in the study of literature) and the natural scientific study of the brain is not overcome, but rather exacerbated by neophenomenology's facile assertions which, in fact, cannot be supported by human science, natural science or phenomenological approaches (see Figure. 1).

Despite claiming that the relationship is not "quantitative" in that the concepts are too "holistic" and inter-related, Sass and Parnas nevertheless contradict themselves in proposing, by virtue of the very words they use, a quantitative relationship between *decreased *or *diminished *self-affection and *increased *reflexivity (hyper-reflexivity) or reflective self-awareness. That is, they propose a standard inverse correlational relationship. However, the increased or *excessive *reflective self-awareness is pre-reflective, or passively automatic, like ipseity itself. Since ipseity pervades pre-reflective self-consciousness and is thus not dissociable from consciousness, it is itself virtually indistinguishable from its compensatory operative hyper-reflexive response. We find ourselves in such a quagmire of nebulous terms and concepts that amorphously flow into one another. The possibility of being able to operationalize and *measure *these constructs according to neuroscientific methods is prohibited from the outset.

Sass and Parnas [79] write: "hyper-reflexivity and transformation of ipseity may ...not be best conceived as outcomes or indices of distinct processes, but as aspects of a single whole ...Indeed it may be argued that these two disturbances are really one and the same phenomenon..." (p. 79). This failure to conceptually distinguish parts of a process which could be defined precisely, remain stable, and eventually be operationalized is indeed a recipe for somewhat esoteric terms, which are difficult to understand and operationalize. *The terms do not seem to have any direct applicability in the understanding, research and treatment of patients who suffer from a very real disorder*. Moreover, since these concepts remain sufficiently vague, they may always be reformulated to include those aspects considered lacking by critics. The authors could offer the counter-argument, "Mishara misunderstands, this is what we meant all along." As a result, they present a moving but also ultimately, amorphous target (see Mishara [6,71]). By *abstractly *and *reflectively *separating "quality" from "quantity" in a manner which does not appear in the original phenomenological experience (see von Weizsaecker, [53]), Sass exhibits some bias to elevate the qualitative over the quantitative and ultimately, may fail to be able to reintegrate these assumptions back into scientific study. This is reflected in numerous statements; for example, Sass writes: "...the characteristically schizophrenic abnormalities of experience defy any simple quantitative description and demand a richer and more qualitative set of concepts" (see Mishara [71]). By restricting their assertions to the qualitative, neopheomenological adherents are always able to make the counter-claim, "well so and so misunderstands because he/she does not have the intuitive capacity and/or background knowledge to grasp the subtle nuances of what we mean." Sass repeatedly states that it is very difficult to describe ipseity which remains a nearly ineluctable concept [71], and this I find makes the term and its use problematic.

In this regard, we might ask how does the prefix, "hyper" clarify the presumed mental processes under investigation? One meaning of hyper is excess, beyond the normal. One could argue that subjects who engage in reflective self-focus do so *more *than healthy individuals or do so *excessively*. But this is not what is meant. Hyper is not simply an incremental "more." Rather, I maintain that it means that the process itself is somehow pathologically increased, *not in the sense of frequency or quantitative intensity, but rather "qualitatively," *i.e., somehow in terms of the internal dynamics of the process itself and whatever underlying pathology would give rise to this "excess."

Following my [80] critique of his concept as not taking into account the more bottom up approach of Binswanger to disrupted self in schizophrenia in terms of Husserl's concept of passive synthesis (see Mishara [19,80,81]; but see also the subsequent discussions [1,48,82]), Sass reformulated his "hyper-reflexivity" concept to include a more tacit, "operative hyper-reflexivity." However, I feel that this adjustment does little to address the initial problem of being unable to provide an account for the bottom up disruption of pre-attentive binding processes of self in schizophrenia (see [2,3,6,71,80]).

Given this situation, we might ask how might we justify the same concept, operational-hyper-reflexive-ipseity, to mean both intensified self-consciousness and "a more operative, automatic, pre-reflective hyper-reflexivity"? Do not automatic and pre-reflective contradict the reflectivity of an increased self-consciousness? The "pre-reflective-operational-hyper-reflexive-ipseity" is the case of a construct which has been so expanded, in its efforts to elude criticism, that it refers to opposite and contradictory phenomena.

Contrary to the promissory, neophenomenological claims that one day we will able to find the neural correlates for ipseity and (operative) hyper-reflexivity, there is not one foreseeable neuroimaging experiment, at least to my mind, that could test such hypotheses. (That is, foreseeable at least to me. I welcome my colleagues to attempt such an experiment. However, in doing so, they should be assured that I, and other members of the philosophic and neuroscientific communities, will be very careful to examine whether they are actually measuring the construct(s) they claim to be measuring. After all, that is the minimal criterion for doing science concerning the kind of bold claims they have been making up to this point). This is because the constructs remain too vague and expansive to be operationalized in terms of our current technologies of measuring cognitive and neural functioning. Ipseity is nearly coextensive with consciousness itself, and hyper-reflexivity, as we are to understand from the authors' own remarks, is nothing but another form of ipseity and ultimately inseparable from it.

Neuroimaging experiments require the constructs under study to be somewhat manageable, i.e., composed of components which are at least dissociable from one another or from something as broad and omnipresent as consciousness itself. They further require the ability to devise a control task which putatively reproduces the experimental task in all its details but with the one construct of interest removed. In this way, the control task may be subtracted from the experimental task to see what remains above and beyond baseline "default mode" functioning of the two groups under comparison. For a discussion of 1) abnormal default mode baseline network activity in schizophrenia, including findings from our own lab; 2) why mere subtractions of the experimental task from baseline may be misleading in neuroimaging experiments with schizophrenia patients, and 3), the difficulty in making any inferences about self-experience or other cognitive activity in schizophrenia from our own finding of abnormal default mode network functioning, see Mishara [6,71]. As a result of these concerns, it is not at all clear how the two constructs, ipseity and operational hyper-reflexivity, could be operationalized in a neuroimaging experiment to ascertain, as the neophenomenologists boldly promise, their "neural correlates.

An additional problem is that the concept hyper-reflexivity and its variant operative hyper-reflexivity do not, even remotely, map onto any known cognitive, neuropsychological or neuroscientific concepts. This would be pardonable if the concepts were sufficiently defined or precise to be operationalizable in their own right, and therefore could be tested, for example, in a neuroimaging experiment. After all, it is very hard to envision (as well as test) an automatic, pre-reflective (non-conscious, and thus not aware) hyper-reflexivity, which is nevertheless by its authors' own definition, an excessive self awareness or which "takes itself or some aspect of itself as its own object of awareness." What would such an operative or tacitly functioning hyper-reflexivity look like which would be both excessive awareness and non-conscious or "automatic"? What would this be if not pre-attentive bias which is well known to the literature, but, being nonconscious, automatic, and unmonitored, would have nothing "hyper" about it? If hyper-reflexivity precedes or is simultaneous with the pop-out it supposedly "explains," *how does it know in advance where to look *(i.e., without recruiting the *Gestalt-kreis *of ongoing switching between egocentric and allocentric coordinates of motor and perceptual selves, respectively, see Mishara [3,25,71]).

It is interesting to note that the neural correlates for attentional networks overlap with those frontal parietal networks recruited for eye-movement (see [25]). Such attentional networks are presumably implicated in the neophenomenologic hypothetical excessive or exaggerated self-awareness, that is, to the extent that their hyper-reflexive-operative-awareness includes attention, a point in relation to which the neophenomenologists have up to till now remained silent. That is, attention itself - just as any bodily self-experience involves the mediation of what I have been calling a Gestalt-circle (*Gestaltkreis*), or a "hidden" and mutually exclusive unity between perceptual and motor selves. This provides counter-evidence to Henry's proposal of the *absolute unity *of bodily self as *immediately *given in the subjective immanence of self-manifestation, and the neophenomenological appropriation of the Henryian exclusively *subjective or inner *body as the "passive" autoaffection of ipseity. There is no *immediate *and pre-reflective access to self which does not have to traverse the dilemma of being both an agentic "I" and a bodily "me" as object, and therefore, the accompanying transform of their respective reference-frames (see [2,3,9,25,48], and below).

As I introduced earlier in this paper: The experienced body (and implicated neural pathways) is comprised by both a motoric-body (proprioceptive-vestibular body-schema), the "I" (as agent), and perceptual-body (exteroceptive body-image), the social "me," united *intermittently, provisionally *and *fragilely *by an interoceptive body (the "mineness" of this relationship) in pre-attentive "efferent" binding of subcomponents of self prior to the emergence of self as a "unitary" experience in awareness [3], p. 609. "Mineness" is disrupted in hypnagogic hallucinations of a double or *Doppelgänger *(thus, contra Zahavi, providing one of many possible dissociations between mineness and consciousness (see [3,71]), i.e., consciousness, even embodied consciousness, may occur *without *an accompanying, but also *occasional *interoceptive-afferent information about self). With regard to the body schema/body image concept, I have written the following summary: "For Paillard [83], the distinction body-image/body-schema is that between 'a conscious awareness of one's own body' and 'a nonconscious performance of the body': 'Proprioceptive information is obviously necessary for updating the postural body frame (or schema), whereas exteroceptive multimodal information, mainly visual, underpins the central representation and percept of the body image...' (pp. 197-198). The body-schema provides a 'path structure', superimposed on a collection of separate points, in a vectorial map which defines in *egocentric *terms how awareness is able to shift from a current 'here' to an anticipated but still not consciously known 'there.' Paillard [83,84] acknowledges the overlap of his model with Milner and Goodale's [85] proposal for a 'vision-for-perception' ventral system which is more recently evolved (mediating awareness) and the more ancient (nonconscious) 'vision-for-action' dorsal system. For Milner and Goodale, the dorsal stream projecting from primary visual cortex to the superior parietal lobes is a key component in an action pathway of visual processing which locates 'where' a relevant stimulus might be in the periphery relative to current focal vision... Information may be relayed to the dorsal or ventral pathways based on its peripheral or central location in the visual field. *Information from the peripheral visual field has faster access to the implicit body-centered computations of dorsal processing streams than the slower ventral pathways subserving conscious focal awareness*. Nowak and Bullier [86] coined the term 'fast brain' for the fronto-parietal connectivity of the dorsal pathways which, according to the Goodale Milner model, mediate implicit visuomotor control (as well as sensori-motor transformations from other sensory modalities necessary for this control). That is, information coming from the peripheral visual field 'has access to fast, direct pathways that allow for faster onset times in dorsal stream areas.' Moreover, we may conclude that the function of frontodorsal connectivity is the 'monitoring of peripheral stimuli in general.' (Stephen et al. [87], p. 3072)." [2], pp. 718-9.

Remarkably, such a system of self as prospective openness, i.e., the ability to be affected by any point in its experiential field (structured by momentary, possible movement) prior to focal awareness had been anticipated by Husserl (Mishara [25]). The location in the field is *prospectively *structured by the "kinaestheses" of ocular motor response, i.e., by a potential field that is structured (nonconsciously) in terms of possible movements (e.g., eye-centered coordinates) required to reorient optimally to the novel target (Claesges, [88]; see also [2,25]). That is, what Sass [86] describes as the pop out resulting from operative hyper-reflexivity is generally described, however, as a top down search in the experimental cognitive literature but there must also be *a bottom up emergent affective contrast saliency which attracts the I-awareness *[67,68]* in which the "I" (motor self) already knows how to get there before orienting*, e.g., the egocentric pathstructure of the eye-movements prior to awareness. The ipseity/operative hyper-reflexivity constructs, with their claims of immediate givenness of (immanent) autoaffection in the subject's naïve pre-reflective self-awareness, do not allow for the *mediation of body as **both **inner and outer *in the emergence of meaningful experience and are unable to address, with their apparently impoverished and vague concepts, the complexity of brain function implicated in the phenomenology of bodily self. This problem, however, deserves fuller treatment (see [71]).

Surprisingly, this debate, at least in its rudiments, has already taken place. The French philosopher, Janicaud [89] criticizes what he calls the "theological" turn in the new French phenomenology (including Henry's concept of pre-reflective self-awareness as ipseity). As the earlier phenomenologist, Jean Herring expresses it, once you allow what is considered phenomenologically given to expand to include metaphysical, biblical, theological, and mystical terms, the rigorous methodology of phenomenology declines into a "pseudophenomenology." "It is not difficult to foresee that the hour when [phenomenology] will become *à la mode *...[and we] will see the springing forth of a whole pseudophenomenological literature." (cited by Prussak, [89], p. 4, insert is mine). It appears to me that this time has come.

Janicaud and then later, Derrida critique the tendency of the new French phenomenologists to expand the phenomenological concept of givenness (e.g., Henry's absolute knowledge of self as prereflective passive self-affection) beyond what is accessible according to phenomenology's own method. Prusak writes: "such givenness is finally a mere concept without intuitions, a concept that it might be possible to think, but that is impossible to experience and to know" [89], p. 4.

As I deal with these themes in more detail in other contributions (Mishara [6,71]), I limit the current discussion to aspects relevant to our main question: to what extent does the operative hyper-reflexivity construct help us understand hypnagogic hallucinations as documented in Kafka's writings, or related phenomena in schizophrenia (see [48,49])? In this regard, Sass [90]*appropriates *Bleuler's concept "doublebookkeeping" as the schizophrenia patient's ability to function in both the everyday world shared with others, and the world of the delusions and hallucinations. For example, a delusional patient claims that she is being poisoned and yet, continues to eat the hospital food. Sass explains that the patient does not act on the delusions and hallucinations because they are "felt by the patient to exist only 'in the mind's eye," that is, what Sass calls the hyper-reflexivity of "an Apollonian illness." Citing Sechehaye's *Autobiography of a Schizophrenic Girl *[91]), Sass [90] defines this Apollonian illness: "... many schizophrenic patients describe the world of psychosis as a place not of darkness but of relentless light - light being the natural metaphor for conscious awareness... [and then citing Sechehaye] 'where reign(s) an implacable light, blinding, leaving no place for shadow,'" p. 117 (my insertion). Sass writes: "In my view, the experience of many schizophrenic patients involves not an overwhelming by but a detachment from normal forms emotion and desire, not a loss but an exacerbation of various forms of self-conscious awareness" [90], p. 12. It is hard to imagine how such Apollonian "relentless light" could become automatic or unconscious in so-called operative hyper-reflexivity when it is a metaphor for conscious awareness itself.

Sass [90] continues that in such an illness, there is "not an overwhelming by but detachment from the instinctual sources of vitality, not immersion in the sensory surround but disengagement from a derealized external world, not stuporous waning from awareness" (p. 117). In contrast, I have proposed that Berze, Conrad, Binswanger, Blankenburg, Ey, Straus and numerous other psychiatrists in the phenomenological tradition (with regard to whom Louis Sass claims, incorrectly I believe, direct lineal descent) describe schizophrenia completely differently as a *Dionysian *illness [1,80,82,92]. For example, the phenomenological psychiatrist, Conrad [93] characterizes the paranoid delusional patient in *a world between waking and sleeping*, "a world of fluctuating Gestalten, concerning which up to this point, the poet has much more knowledgeable things to say than the psychologist" p. 378, my translation. As schizophrenia for Conrad is a being "caught between sleep and wakefulness," double bookkeeping is not some intellectual indulgence, an *intensifying *of intact *rational *or *attentional *processes, an ability to detach and participate willy-nilly in two worlds by straddling them. Rather, as in the disorder sleep paralysis,^xlii ^the patient is simultaneously aware of two "realities," the compelling reality of her hypnagogic hallucinations or felt presences (*from which the patient is unable to critically detach*), on the one hand, and the world of her awake life on the other [3,48]. *Caught somewhere in between, it is not that she belongs to both worlds but to none, and tries unsuccessfully to find her way back*.

By comparing the experiences of schizophrenia with sleep paralysis, I am not endorsing the so-called "rapid eye-movement (REM) hypothesis" of schizophrenia, but rather that schizophrenia, like dreaming, hypnagogic or autoscopic hallucinations involves a disengagement or *reduction *of what cognitive neuroscience calls conscious controlled processing, *not *its exacerbation (whether reflective or pre-reflective, controlled or automatic, top down or bottom up, or whatever other variant of his hyper-reflexivity concept Sass wishes to claim). Experimental and neuroimaging results with schizophrenia patients decidedly do not support the "Apollonian" interpretation (see [3,6]). Note that the sleep paralysis patient, as the psychotic, may feel in her state infinite distance from the world shared with others (what we have previously described as social deafferentation, Hoffman [34]). Moreover, the feeling of a presence,^xlii ^or the intensity of hypnagogic hallucinations, as we have seen above, may be a response to this feeling of distance or deprivation (and the underlying neural processes). This is not the detachment from too much intellect or lack of emotion. It is rather precisely *the inability to distance*, or separate from (i.e., transcend) what Conrad [93,6] calls the physiognomic expressiveness of the hallucinatory "pre-Gestalt" (*Vorgestalt*) during paranoid psychosis and hypnagogic experiences. Moreover, it is not the detachment resulting from too much intellect or lack of emotion, a hyper-reflexive "*solipsism*" [90], see [71] for review. *It is rather precisely the inability to critically distance, separate from (i.e., transcend) the physiognomically expressive Vorgestalt, in an oneric "world" populated by hallucinated doubles* (i.e., precisely not a solipsism but a modified intersubjectivity [71]). That is, delusions and hallucinations form very much like the experience of objects in a dream or in early stages of perceptual meaning in tatiscopic experimental studies of microgenesis. These incomplete "objects" (taken as if they were objects complete with perceptual meaning) are very much like the hypnagogic doubles described in Kafka's writings and those hallucinations experienced in autoscopy [3].

Conrad describes the patient's subjective experience of delusions (e.g., "delusional perceptions, "ideas" of reference) as a "protopathic functional change of the pre-Gestalt (*Vorgestalt*)," (employing Sir Henry Head's and von Weizsäcker's [53] concepts respectively; for reviews of Conrad's use of Head's and von Weizsäcker's terminology, see [48,49]). In contrast to the neophenomenologic imprecise analogies between their concepts and contemporary neuroscience (see [71], and above), Mishara and Corlett [97] provide a neurobiological account of Conrad's phenomenologic approach to aberrant saliency in beginning psychosis in terms of the disrupted prediction error of a perception action cycle.

Sass (e.g., [90,98,99]) emphasizes that his own analysis based on Sechayes' [91]* Autobiography of a Schizophrenic Girl *is consistent with Conrad's [95] phenomenological approach. We have already noted that Sechehaye's book is central to Sass' own Apollonian hyper-reflexive account of schizophrenia. However, Conrad [95] is very clear in his classic monograph in devoting over five pages (!) to refuting any effort to examine schizophrenia, which bases itself on Sechehaye's book, denouncing the *very same *passages which Sass [90,99] cites as evidence for the harmony between Conrad and his use of Sechehaye's work (for complete discussion, see [71]). In short, I maintain that Conrad's concepts do not support the Apollonian or "operative" versions of the hyper-reflexivity concept (as further documented in other publications, e.g., [71]). The inability of the schizophrenia patient to shift perspectives or frames of reference in terms of a *Gestaltkreis *is central to the works of Binswanger, Blankenburg, Conrad, Kraus, Wyss and numerous other phenomenologic psychiatrists [48,49].

It is striking that those proponents of what I have labeled the neo-phenomenological position *completely *omit Viktor von Weizsäcker's work or his concept of *Gestaltkreis *from their discussions. This is remarkable because von Weizsäcker's work has been acknowledged by many of those phenomenological thinkers and clinicians - cited by the neophenomenologists as the very phenomenologists supporting *their *position - to play an important, if not major role in the development of their thinking, e.g., Binswanger, Blankenburg, Bujtendijk, Conrad, Ey, Merleau-Ponty, Plessner, Plügge, Straus, and countless others. In fact, I have had numerous conversations concerning von Weizsäcker's influence on phenomenology (including their own work) with Blankenburg, Bräutigam, Christian, Claesges, Gadamer, Graumann, Hahn, Janzarick, Jantz, Kraus, Kuhn (Binswanger's close friend and discoverer of Imipramine, the first tricyclic antidepressant), Landgrebe, Lang, Mundt, Tellenbach, von Bayer, von Uslar, T. von Uxkühl, C.F. von Weizsäcker, R. Wiehl and Wyss and others. *The neophenomenological neglect of von Weizsäcker's work is egregious but understandable, given the extent it undermines their own position*.

In keeping with the current effort to examine the literary "data" concerning paranoid hypnagogic experience, Conrad characterizes the paranoid delusional *world between waking and sleeping *as "a world of fluctuating Gestalten, concerning which up to this point, the poet has much more knowledgeable things to say than the psychologist" [93], p. 378, my trans). Conrad observes the similarly between earlier stages of unformed perceptions in microgenetic experiments, or impoverished perceptual conditions, and the dreamer's or hallucinating patient's acceptance of the incomplete Gestalt (*Vorgestalt*) as given. In describing the application of microgenetic procedure to schizophrenia, Flavell and Draguns [100] describe "schizophrenia as a condition in which early cognitive formations intrude into consciousness and get expressed as though they were completed thoughts." Once we are able to detach from the incomplete perceptions and our productive responses, e.g., hallucinations, dreams or even literary creations, *they release us *and suffer a kind of *death *(Conrad [96], p. 41) as when the child ghost releases the narrator in Kafka's *Unhappiness*.

Broad and sweeping general statements have been made about Kafka and "modernism." For example, Kafka, has been called "the representative writer of our century" (Karl [101], xvii), but, given Kafka's documented proclivities for social isolation, we ask *representative for whom*? Similarly, Sass [98] calls Kafka "a figure representative of the age," and "one of the most representative of twentieth century writers." Nevertheless, Sass [98] also makes the highly controversial remark (which I examine elsewhere [71]) that Kafka presents "the most vivid evocation of schizophrenic experience in all of Western literature." That is, according to Sass, Kafka is both modern (representative of the age) and depicts the schizophrenic condition. He is characterized by Apollonian "detachment" and "indifference," which, for Sass, is shared by both modernism and schizophrenia. Unlike Sass, I make *no *effort, apart from Kafka's possible cluster headaches [15], to diagnose Kafka on the basis of his literary work. However, to the extent that we consider the problem according to Nietzsche's [102] Apollonian vs. Dionysian opposition, there is no doubt on which side of this dilemma Kafka himself stands. Kafka writes, "What are you building?-I want to dig a subterranean passage. Some progress must be made. My station up there is much too high. We are digging the pit of Babel" (Kafka [10], p. 464). That is, building = writing (as in *The Burrow*) cannot be achieved from the lofty heights of reflective awareness (as we have seen from Kafka's *The Bridge*). There is no reflective distance here. Kafka's preoccupation with the self's *depths *(as already suggested by the symbolism of the mirror in his story, *Unhappiness*) was not born from Apollonian, detached reflection of hyper-concentration, what Merleau-Ponty [78] describes as an "aerial perspective." It was also not born from a putatively buried, tacitly functioning, but also neurobiologically implausible "operative" hyper-reflexivity. Rather, Kafka describes his own writing as *a Dionysian descent in which the self becomes dissolved in its own origins *(i.e according to the symbolism of rebirth which the "twice born" Dionysus himself experienced).

For Kafka, writing is a *trancelike *Dionysian activity at night opening the endless inner darkness of self as an abyss without bottom. Recall Kafka's own descriptions of the writing process, e.g., "From the depths I would drag it up! Without effort!," or, "All I possess are certain powers which, at a depth inaccessible under normal conditions, shape themselves into literature..." or that it is "not alertness but self-oblivion [that] is the precondition of writing" [41,46], and cited above. Here, self-depiction in art hardly takes an Apollonian turn (in the sense of Sass' concept of hyper-reflexivity) [1]. In Kafka's stories, "*A Dream*," "*A Hunger Artist*," and "*The Burrow*," digging into, or merging back into the earth exert an insuppressible attraction on the protagonist. The burrowing into earth, the (endless) inner journey of the self as underworld in the short stories, *Hunter Gracchus, A Visit to a Mine*, or *I was a visitor among the dead *is a Dionysian attempt to access the self from inside in terms of its inner depths (Kurz [14]; Mishara [68]), not the Apollonian perspective of the detached aerial view of hyper-reflection. For reviews of the Dionysian critique, see [1,80,92].

## The Hypnagogic Symbolism of Rebirth: How the Self reflects its Own Structure as a Process

In his novel, *Amerika*, Kafka depicts his protagonist, Karl Rossmann as scribbling in his notebook with a fountain pen the manageress had just given him. In itself, this event is not particularly remarkable. However, as Malcolm Pasley [103] notes, "Shortly before writing this passage, as one can see from the manuscript, the novelist clearly had to struggle with, and replace, a malfunctioning fountain pen. Here we see how closely the written text - the work - relates to the physical act of writing and even to the small calamities that can occur during the writing process" (p. 203). Now, this is rather an odd breaking of the narrative frame to include an event, which occurs during the physical writing itself, but which seems trivial in its own right. However, we see numerous instances in Kafka's narrative texts which include references to Kafka's own writing or artistic activity during the very composition of the text (e.g., the writing desk in "*I was a visitor among the dead*," the artist's pencil which produces in gold lettering the letter K on the protagonist's own tombstone while the protagonist watches in *A Dream*,^xliv ^the punitive writing machine in *A Penal Colony*). Here, the act of writing seems to be reflected in the symbolic imagery of the narrative itself.

**Figure 4:**
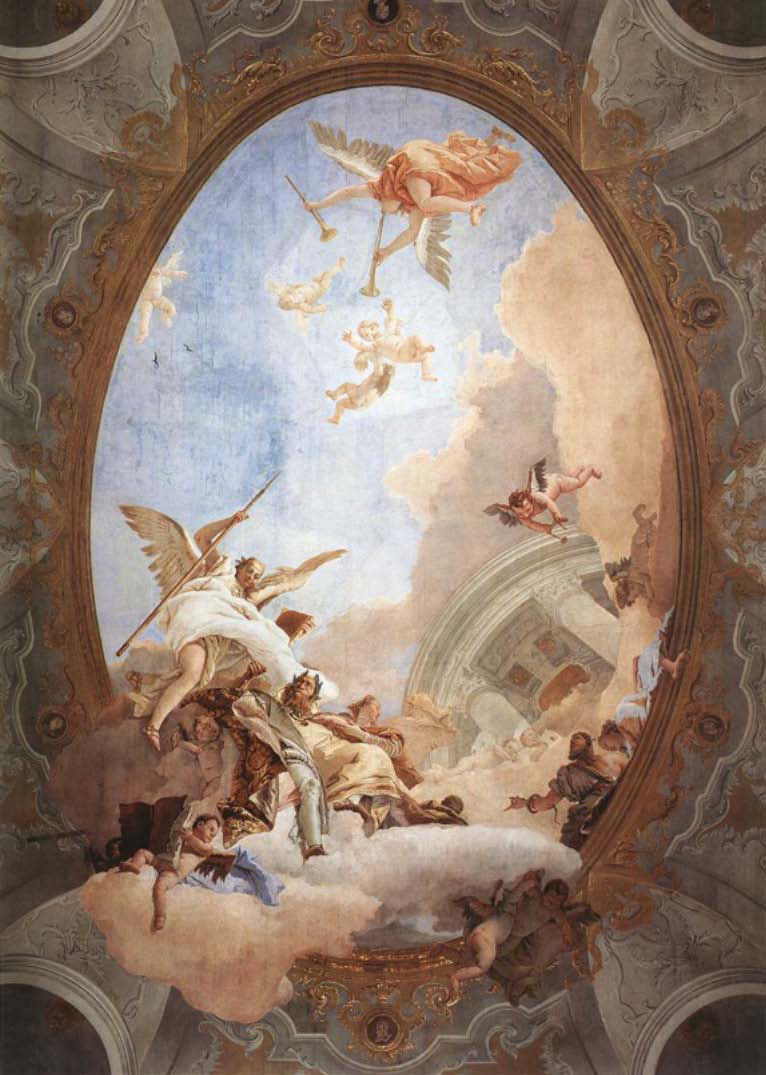
**Example of Rococo Art Breaking Frame, Phillip Friederich von Hetsch's *Allegory of Merit Accompanied by Nobility and Virtue *(1758)**.

We know such "breaking frame" from Baroque rococo paintings (e.g., [104]). In figure 4, the artist, Phillip Friederich von Hetsch, deliberately portrays his figures as actually descending towards the viewer from the frame of the architecturally spatial area meant to contain them. However, this deliberate device only remotely resembles what is going on in Kafka's work. When Kafka includes the reference to Karl Rossmann making use of a lent fountain pen in *Amerika*, it is clear that he is not expecting his audience to know that he himself as writer had to just replace his own malfunctioning pen. That is, *it is likely that the initial occurrence of the symbolically reflexive meaning (Silberer's auto-symbolism) in the flow of hypnagogic images that putatively informed Kafka's writing was initially unconscious*. As already noted, Kafka himself reports that he experienced writing (at least in its initial phases) as automatic, effortless and informed by hypnagogic imagery. That is, Kafka both records and shapes his hypnagogic imagery and it's meaning while writing in what I have described as a trance-state during sleep-, but also sensory- and social-deprivation. Kafka's spontaneous self-reference to his own writing activity in the symbolic imagery of the hypnagogic imagery he records while writing *initially *functions precisely to the degree that it remains nonconscious, i.e., not directly formulated in awareness, without so-called Apollonian hyper-reflexivity playing a role.

As noted with Silberer's introspective experiments regarding his own hypnagogic experiences while falling asleep, *a requirement for the autosymbolic hallucination to occur, the subject is unaware at the time that his own mind is producing its symbolic meaning*. The phenomenological psychiatrist, Conrad also reports similar introspective results with regard to his own hypnagogic imagery. He states, "precisely in the relaxing of effortful attention, indeed in passive dreaming sunkenness, the solution appears in an image. *It is important to note however that in the dream it does not appear as a solution to a long sought after problem but rather as an imaginary presenting of the sought for relationship forming indicidentally*. It is only in waking that it is realized that the image brings a solution to the puzzle" (Conrad [96], p. 39, my translation and emphases). That is, as we have already seen in the dilemma of the narcoleptic sleep paralysis patient, who, like the country doctor, is caught between worlds, the two worlds, conscious waking experience and the formation of autosymbolic hallucinations are mutually exclusive and yet, one "world" is unable to exist without the other. The phenomenological psychologist, von Uslar [105] writes of two mutually "excluding realities of the waking and dream worlds" (p. 19). These two worlds mutually presuppose but exclude one another in the dialectical relationship of a Gestalt-circle.^xlv^

Nevertheless, as we have already noted, there is something about the process of the self-reference of the writer in Kafka's hypnagogic protagonist doubles that remains incomplete. In Jungian terms, the inner "journey" of symbolic transformation of self as a process of rebirth through narrative imagery remains unfinished. We find a hint in Kafka's remarkable story, "The Hunter Graccus" (1916/1917). Here, the protagonist Graccus (whose name we have already noted suggests Kafka's own name) is unable to die. Generations and generations of bureaucrats are unable to arrive at a decision before being interrupted by their own deaths. As a result, Graccus' leaving this world has indefinitely put on hold . *Due to fact that the character of Hunter Graccus is already fictional, one might argue, taking a rather literal standpoint, that he is unable to die because he has never lived*. He actually recounts his life as fictive in the following (deliberate) "slip" that Kafka inserted into the text: "Nobody will read what I write here."^xlvi^

That is, it is no longer the fictive Graccus speaking but Kafka as narrating author, who once again bemoans the incompleteness of his narratives now depicted in Graccus' infinite journey (caught between worlds), without foreseeable end. Similarly, the protagonist at the end of *A Country Doctor *complains of a journey without end: "Never shall I reach my home at this rate. ...Naked, exposed to the frost of this most unhappy of ages, with an earthly vehicle and unearthly horses ...My fur coat is hanging from the back of the gig, but I cannot reach it" [10], p. 225. In still another story with a similar ending (i.e., without end), the protagonist in *The Bucket Rider *finds himself exposed to the *cold *without any promise of ever finding an end to his journey. Here, the protagonist has the unique ability to ride his bucket through the village precisely because he has run out of coal and the bucket is empty: "I must have coal; I cannot freeze to death; ...Seated on my bucket, my hands on the handle, the simplest kind of bridle, I propel myself with difficulty down the stairs, but once downstairs my bucket ascends superbly, superbly" (pp. 412-3). While hovering outside the coal-dealer's window and appealing to the latter's wife for some coal, he describes his experience: "She sees and hears nothing; but all the same she loosens her apron strings and waves her apron to waft me away... My bucket has all the virtues of a good steed except powers of resistance, which it has not; it is too light; a woman's apron can make it fly through the air... And with that I ascend into the regions of the ice mountains and am lost forever." In the bucket rider, Kafka [10] depicts the feeling of a passively floating out of body-like experience which occurs as hypnagogic hallucination during sleep paralysis and/or dreaming [3], but still, unlike dreaming, from which we can wake, these stories refer to their own incompleteness or absence of any resolvable ending.

The journey to the interior of the self as endless descent to an underworld is suggested by Kafka's stories, "A Visit to the Mines," and "Hunter Graccus." It is also reflected in contemporary existential thought. Jaspers had compared inner reflection to a conversation or a journey in which one cannot predict one's destination from one's starting point. The discovery of the inner as boundless and dangerous is also reflected in Jung's work. In the 1960's, the metaphor of inner journey as a process of rebirth of the self was sometimes used in the experimentation with psychedelic drugs. The phenomenological psychiatrist Wyss [106], who had trained with both Jaspers and von Weizsäcker, writes: "Self-perception, the inner world appears primarily dark... In self- perception, there emerges from the darkness, 'from out of the depths', phenomena such as memories, images, thoughts, moods, which enter and disappear from the horizon of waking consciousness as a 'stream of consciousness'" (pp. 166-7, my translation).

As I have previously written, "During the narrative effort, the self simultaneously takes on the roles of the narrator and the narrated self of the traumatic event. The process of separating these selves, letting go, and sense of completion is still under way.... Straus wrote that we experience distance not in terms of objective space but in terms of our own momentary ability for movement. It is for this reason that we have no distance in the dream because the dream landscape moves with us and encloses us within its horizon. We are always in the present in the dream, enveloped within the immanence of our own bodies, in a private universe" (Mishara [18]). Thus the ability to seamlessly shift frames between a narrating subject and one which is embedded within the development of the narrative may have its (neural) basis in the opposition body image/body schema. To narrate about oneself is an act of self-transcendence because one experiences oneself as *both *active narrating subject *and *narrated object (as if seen from others' point of view). This reflects a fundamental paradox of human existence, I can only become myself by letting myself go, by transcending what I just was (von Weizsäcker [53]). For Sartre [39], I am divested from my body as object the moment that I assume it. That is, I can only *have *a body in retrospect, but am condemned to *being *a body self prospectively open or vulnerable to its own future who, at each moment, must sever itself from (i.e., transcend) each of its ongoing achievements/experiences as already past [25].

I propose that the *symbolic *doubling process recorded in Kafka's narrative imagery reflects something deeply embedded in how the human capacity for narrative evolved, in the relationship between the author's, or narrator's self and that which he/she narrates. That is, *the act of narrating requires an ongoing shifting between internal and external perspectives or reference frames with regard to one's own body*. The narrating I is egocentric in that, like movement, the next steps in a narrative is computed in egocentric coordinates. *However the narrator narrates a self already embedded in scene based allocentric coordinates and becomes entranced like his audience in the unfolding of these scenes from the standpoint of the protagonist*. The narrator must shift back and forth from being absorbed in the narrative unfolding of his/her own story to quick decision processes of "where" to go next, which narrative path to take, not unlike envisioning chess moves, or taking a new route, several steps in advance. The latter process occurs in terms of what I have been referring to as the egocentric reference frame of an on line body schema. The double (as in Kafka's initially hypnagogic narratives) symbolizes the embodied self precisely as a process which in its very nature is a self-transcending [2,3,18,39,53,62,67,68,78,95].

## Conclusions

In summary, the structure of the self is vulnerable to doubling. This doubling occurs during anomalous states of consciousness (where reflection is minimal and trance-like, dream-like single-mindedness prevails). It involves the structure of self as both self-transcendent and intersubjective (in terms of the symbolic imagery of "rebirth"). The literary data considered in this paper reflect a change of consciousness which has its neurobiological basis in increased cortical excitability of a social network (activated during states of deprivation, sensory, social and sleep). The paranoid delusions of schizophrenia resemble hypnagogic hallucinations in the patient's inability to detach from incomplete information, what the phenomenological psychiatrist, Klaus Conrad describes as a pre-gestalt (*Vorgestalt*), and what has more recently been called, "aberrant salience." Current neuroscience studies the self as object or representation. Literature informs the clinical neuroscientific study of self because the structure of self (as self-transcending process, i.e., as both subject and object) is captured in a literature which reflects its own creative process in hypnagogic symbols. Contrary to the apparently popularizing, but unverifiable views which interpret the self as "distorted" pre-reflective, hyper-reflexive self awareness in neuropscyhiatric disorders and anomalous conscious states, *the "self" informs our hypnagogic imagery precisely to the extent that we are not self-aware*.

## Notes

i. This paper is much modified and expanded version of a paper, "The Literary Neuroscience of Kafka's Hypnagogic Hallucinations: How Literature Informs the Neuroscientific Study of Self and its Disorders," to appear in Jaen-Portillo, I., Simon, J. (eds). *The Cognition of Literature*.

ii. Phenomenological-psychiatrists and researchers (e.g., Binswanger, Buytendijk, Tellenbach) justify their use of literature as a source for phenomenological "data" for providing the structures of healthy (and abnormal) consciousness by referring to Husserl's method of imaginative variation (described below).

iii. In Kafka's work, the writer's self is doubled in the protagonist in different ways: 1) The narrator's and protagonist's perspectives collapse into one another. That is, the narrator's purview is limited to that of the protagonist so that we experience everything from the protagonist's claustrophobic view-point (as in Kafka's novels, *The Trial, The Castle*). 2) The protagonist is named some variant of Kafka's own name such as "K" or "Josef K." In Kafka's *The Metamorphosis*, the protagonist's name, Gregor Samsa resembles Kafka's own name (S*a*ms*a *= K*a*fk*a*). The Hunter Gracchus in Kafka's story with the same name also suggests the name Kafka (*Kavka *in Czech means Jackdaw, and *graccio in Italian *means Crow's caw or call). That is, the protagonist stands in for the author as a double, but takes on a life of his own.

iv. Previous commentaries on the "doubles" in Kafka's literary work cite as instances of such doubling either the twin helpers, Artur and Jeremias, in Kafka's *The Castle*, or the two "small white celluloid balls" which seem to pursue *Bloomfield, the Elderly Bachelor*. The latter are later replaced by two assistants who resemble the twin helpers in *The Castle*. Collins [11] interprets these instances of doubles as serving the function of a "chorus, personifying the society that accuses him inexplicably" (p. 7). He suggests that one source of Kafka's use of twin-doubles (in *The Castle *and *Bloomfield*) was a Yiddish theater performance Kafka witnessed in 1911. As indicated by his diary entry at the time, Kafka was impressed by the two twin clown-like figures played husband and his wife ("Mrs. K., a 'male impersonator'"). "Light as a feather, [they] sink to the ground under the slightest pressure...sensitive, [they] cry easily with dry faces ...but as soon as the pressure is removed [they] haven't the slightest specific gravity but must bounce right back up the air" (Kafka [12], p. 65). Flickert [13] interprets the two dancing, bouncing twin celluloid balls in *Bloomfield *in terms of the *Doppelgänger *motif which has been prevalent in German literature since the Romantics. However, these commentators do not take make the additional claim, as I do here, that Kafka's doubling pervades his entire corpus, i.e., that his relationship as author to his own protagonists is itself a doubling process, which is further reflected in the encounters between the protagonists and the figures they encounter. Moreover, the *symbolic *doubling reflects something deeply embedded in how the capacity for narrative evolved in the human brain, i.e., in the relationship between the author's, or narrator's "self" and that which he/she narrates, as both narrator and witness to one's own narration (see final section of this paper). In this regard, Kurz's [14] observation is more relevant than Collins' and Flickert's prior commentaries: "Kafka's heroes are split-figures, split into themselves and others, *their *others. Kafka himself was a split-person, on the outside, a thoroughly correct office worker, who took his obligations seriously, and yet also, someone who is 'nothing else than literature' (from Kafka's Diaries)" (p. 31, my translation). Kafka's frequent mention of a "writing desk" in his fictional writings (see below) suggests both his day job in the insurance office but also his stealthy literary activity at night. Kurz writes that "the protagonist... is the measure of time and space for the other figures. These only exist through his view or in his thoughts." ([14], p. 187). The other figures then seem to have a dependence, not unlike a dream, on the protagonist's perspective. Not unlike a dream, they appear as dependent on the protagonist's perspective "as variants of one another... doubling or tripling oneselves" ([14], p. 188, my translations).

v. The mirror is locus of both self and double.

vi. Clearly, readers not acquainted with Kafka's living circumstances at the time of writing this story could hardly be expected to appreciate this parallel between the narrator and Kafka. We might ask then, why is it in the story? Is it a mere association that Kafka himself had while writing (possibly inadvertent) which he did not subsequently bother to remove despite the fact that most audiences would be unable to appreciate it? Or, is it reminiscent of those architects who built ornate statues hidden high in the corners of Gothic cathedrals for God's appreciation alone as no human eye could see them? In the final section of the paper, I return to this problem.

vii. Doppelgänger (autoscopic) hallucinations may occur in neurologic disorders and anomalous conscious-states (e.g., dream-states). The hallucinated double is experienced (precisely as double) as both stranger and intimate. As a result, there is often a resulting struggle of who "mirrors" whom [3], also present in Kafka's story. The "double" need not physically resemble the self and may even differ in gender. Here, there is the suggestion that a literary or fictional character, who serves as a double for the writer himself, may herself entertain questions about her own existence (see the discussion of *The Hunter Gracchus *below). As we will further examine, "fattening up" the existence of the fictional character may be contrasted with its complete dissolution as in *The Hunger Artist*, who, as *artist*, refers to his own status as (symbolic) double of the author. "Fattening up" such an existence is contrasted with its complete dissolution as in "The Hunger Artist," who, as *artist*, refers to his own status as (symbolic) double of the author.

viii. Autoscopy (from the ancient Greek, 'seeing oneself') is a loosely related complex of experiences in which one sees or experiences a "double" as external to one's current vantage point. These hallucinations may occur in epilepsy, brain-tumors, schizophrenia, depression, intoxication, dissociative experiences, hypnagogic/hypnopompic hallucinations and in individuals with high fantasy-proneness. Literary authors often describe autoscopy (e.g., G. D'Annunzio, F. Dostoevsky, J.W. v. Goethe, E.T.A. Hoffmann, G. de Maupassant, A. de Musset, E. A. Poe, J.P. Richter, P. B. Shelley, R.L. Stevenson), many of whom experienced autoscopy themselves [3].

ix. In what is termed echopraxia (imitation of the subject's movements), the double reaches up to turn on the light, which the patient also attempts. However, the double moves her left-arm symmetrically to the patient's as in a mirror. This suggests that the perceptual "body image" is involved. When we look at a mirror we see only the perceptual body (the body as we see it, or imagine it, from outside) reflected in a left-right reversal: when the patient moves her right arm, the mirror-double moves her left. Precisely as perceptual body image, the mirror image is computed in the coordinates of an allocentric, object-centered reference frame. If the double, as it sometimes happens in autoscopic hallucinations, imitates my movements, but moves the contralateral arm to me (e.g., his right arm, when I move my right arm), then the double is engaging, or 'making use of' my motoric-body (moving in the same egocentric, body-centered coordinate reference frame in which I move my own body, i.e., the "body schema" as agentic "I"). This appearance of the double, I argue [3], indicates a "*deeper*," more engaged hallucinatory involvement with the self and therefore, different neural pathways are implicated. The two experiences (and underlying neural systems) of body, one motoric (proprioceptive), the other perceptual (exteroceptive), have been called in the neurologic literature, body schema (the agentic "I") and body image (the social "me") [2,3,22-25]. Interestingly, although more convincing, the motor doubles are much less life-like than the perceptual-mirror doubles and tend to be colorless, pale, transparent, cloudy, misty, or ghost-like. In contrast, the perceptual-body-image autoscopy, predominantly involving lesions to visual-occipital areas of the brain, is generally more vivid and brightly colored. However, the motoric double (often involving parietal and tempoparietal junction areas) is experienced as more convincing than its perceptual counterpart. The view that the more motoric, body schema autoscopy may be delusional (or dreamlike) is supported by the fact that this form of autoscopy can lead to death or suicide through fighting with or trying to free oneself from one's double (an I/I rather then the mirror-like I-me relationship of body-image autoscopy). In a previous publication, I have labeled the more superficial perceptual autoscopy, Type I (predominantly visual autoscospy), and the deeper variant involving the motoric body schema, Type II (delusional-dreamlike autoscopy) [3]. It is only in response to Type II autoscopy that the patient in a confused state may attempt suicide through fighting with or trying to free oneself from one's double. . The case of the mourning schoolteacher [21] described here, however, does not fit neatly into this clear-cut taxonomy of autoscopy. The patient exhibits a disruption of body image in that the double's imitative movements are symmetrical to the subject's with the same left-right reversal that one experiences in a mirror. However, the double also *touches *her in a way that leads to a depersonalizing relationship to the patient's own body (the patient felt "cold and bloodless from the contact"). This suggests involvement of the "deeper," multi-modal engagement of self in the Type II, delusional-dreamlike autoscopy [3].

x. "... war er nur beobachtend, da ich mich eben beobachtete..." Kafka [26], p. 342.

xi. This recalls the above discussion of the two types of autoscopy. If the motoric-body is implicated (in "Type II delusional-dreamlike autoscopy" [3]), the hallucinatory-double takes independence from the self (in an "I/I" relationship) and anticipates the subject's actions. One's own self is experienced as correspondingly *passive *as if the double were usurping or preempting the sense of self as the empowered agent. Unlike the mirroring in Type I (predominantly visual) autoscopy which involves body image, the motoric body schema autoscopy is characterized by the feeling that one (ironically) becomes the mirror image of the double, who usurps the feeling of being the "real self." There may be a feeling of oneness with the hallucination as if the two terms (self and double) are "emotionally linked," share a "feeling of belonging," or complete one another [3]. Brugger et al. [27] depict the experience of an autoscopic patient, PH, who has an invasive tumor originating in the left posterior insula, destructive of his left-temporal lobe, and extending into left frontal and parietal areas. Just as Kafka reports a split relationship with himself vis-à-vis the observing double in the mirror, PH reports awakening one night to find that his *bodily self has split in half*. Concurrently, five "doubles" either mimic or act independently from him on his right side, contralaterally to the tumor. The presence of more than one double is called "polyopic heautoscopy" [27], and is a variant of what I have classified as the more engaging Type II delusional-dreamlike autoscopy [3]. Here the doubles both anticipate the patient's own movements in echopraxia, but also act independently from him. In E.T.A. Hoffmann's remarkable short story, *New Year Eve's Adventure *[28], one of the characters, Erasmus describes how he came to loose his mirror image which he experiences most painfully as a split with himself. Somehow forced to narrate the story in third person, i.e., as now an I/I rather than I/me relationship [3], he recounts how he first lost the image: "Erasmus saw his image step forward independent of his movements, glide into Giuletta's arms and disappear in a vapor" [28], p. 122. As in Kafka's description of the mirror, or PH's multiple doubles, the image acts independently from the self. Once the double acts independently, or *anticipates *the subject's own movements and thoughts in Type II motoric-dreamlike autoscopy, it is as if it steps outside the mirror and begins to *act *in the (normally unconscious) egocentric, body-centered coordinates of body schema [3]. *Moreover, the double has access to the subject's own intentions before or during their execution*. Although Kafka and Hoffmann both describe experiences that initially involve mirror reflection, the fact that the mirror image starts to act independently from the self suggests that the body schema and not merely the more superficial, mirror-based perceptual body image is involved.

xii. The fact that the protagonist "awakens" to find himself in entirely changed circumstances, as in Kafka's *A Country Doctor *(see below) and *The Trial*, suggests that the transformation has somehow occurred or started to occur during the night, while the fictive character dreams, but his author remains awake, sleep deprived, through the night (as was often Kafka's practice during writing). We will further explore below the protagonist's nocturnal "transformation," a "journey" between worlds, as referring, in part, to Kafka's own transformed state of mind while writing.

xiii. "The decision he must disappear was one that he held to even more strongly than his sister, if that were possible" [10], p. 135. Cf. Kafka's *A Hunger Artist*.

xiv. The charwoman is "not allowed to tell her story." [10], p. 138-9. This and other allusions to incomplete or interrupted narratives may reflect Kafka's own experience of the failure of his narratives to connect the subjective-inner experience of self with the outer social self as it is experienced by others (in this case, the "thing" which the Charwoman disposes of). Kafka sometimes narrates his protagonists as caught between worlds, and between mental states, never properly received by an audience (as *A Hunger Artist *(1922) (and Kafka himself) because of the thingness of the body, or the *corpus *of the text which, rather than conveying expressions of one's subjectivity to others, only gets in the way. See the discussion of *An Imperial Message*, below (See also *A Little Woman *(1923) and *First Sorrow *(1922), written around the same time as *A Hunger Artist*).

xv. There are numerous examples in Kafka's writings of autoscopic doubling in which the very mirroring becomes obstructive to the protagonist's own objectives. In Kafka's earliest published story, *Descriptions of a Struggle *(version A written 1903-4; version B, 1909), the narrator's companion (an acquaintance just made at a party) continually does the opposite to what the protagonist anticipates, e.g., he walks too slow or too fast. However (as in the Type II, deeper autoscopy), the narrator suddenly finds himself so embroiled that he catches himself mirroring (!) his companion: "he began walking again and I followed without realizing it..." (Kafka, 1983, p. 13) As much as the narrator desires to escape, he is unable to disentangle himself from the acquaintance as if they were each incomplete on their own, or different sides of the same person. E.T.A. Hoffmann's *New Year Eve's Adventure *[28] may have served as inspiration for Kafka's early story about doubling. In Kafka's *The Castle*, the protagonist, K., ironically experiences an obstructiveness, similar to the companion in *Descriptions of a Struggle *in his twin "helpers," "Artur" and "Jeremias" very efforts to help. As evidence of their status as doubles, incomplete on their own, K. wants to treat them as a "single person" by calling them both by the same name, "Artur." In *Blumfeld, an Elderly Bachelor*, the two celluloid balls appear precisely in the moment of need, or loneliness (as do the doubles in *Unhappiness *and *Descriptions of a Struggle*), i.e., when Bloomfield expresses the wish to have "a companion, someone to witness" his daily activities. However, they do exactly the opposite of what the Bachelor wants and yet, like the deeper Type II autoscopy in which the motoric body schema is implicated, are inextricably connected to the bachelor's own movements: "Suddenly, quite unexpectedly, he [Blumfeld] ...with a jerk he turns around in his chair. But the balls, equally alert, or perhaps automatically following the law governing them, also change their position the moment Blumfeld turns, and hide behind his back." [10], p. 188.

xvi. As in *Metamorphosis*, the protagonist's body (as fictive double of the author) is ultimately destroyed in other Kafka stories (e.g., *The Hunger Artist, In The Penal Colony, The Bridge*). Alternative fates of the protagonist are that he is unable to die, *Hunter Graccus*, or similarly to Graccus, trapped in an unending journey between worlds, e.g., *A Country Doctor, The Bucket Rider *(see below).

xvii. Experimental light-deprivation temporarily induces Charles Bonnet Syndrome (the experience of complex visual-hallucinations, often accompanying visual degeneration). Similarly, subjects who participated in Lilly's "isolation-tank" (sensory-deprivation) experiments (floating in darkness immersed in salt-water at body-temperature) reported vivid hallucinations.

xviii. The phenomenological psychiatrist, Binswanger, for example, writes that the dramatist Ibsen removed himself from others, i.e., sought isolation, to become closer to them. The phenomenological theory proposes, in a manner that the findings of social neuroscience seems to be ever more closely approaching, that the structure of the self ***is ***intersubjectivity, that deep within the self, the self *is *the other but in way hidden to itself [3]. Sartre [39] writes: "what we will discover at the basis of ourselves is others..." (p. 115). Similarly, Kafka describes his own efforts to overcome social isolation "as a return to others by way of peculiar detour" [26], p. 571, my translation.

xix. However, as Kurz [14] observers it is often not clear whether Kafka means that the noise (which he tried to escape) comes mostly from his family or from his own self.

xx. In *The Warden of the Tomb*, the warden addresses the prince with "Du" to which the prince, taken aback, responds, "So we are on terms of intimacy, and yet today is the first time I have seen you." [10], p. 109. Sudden, unexpected, even intrusive gestures of intimacy between the protagonist and other character(s) in the story also occurs as we have noted in "Unhappiness," and suggests that self and other, each incomplete on their own, are inextricably related to one another in (motoric-dreamlike autoscopy, i.e., Type II) autoscopic doubling [3].

xxi. The "ghosts" may, in part, refer to the seductive, or persecuting doubles of his own imagination, as the child ghost (born from loneliness) in *Unhappiness*.

xxii. "I ...on awakening find hanging from my jaws, say a rat, as indubitable proof of night labors which already seem unreal" [10], p. 329). The Muir's translation is misleading and is more accurately rendered as "proof of last ***night's ***work which ***nearly appears as a dream***" (*...fast traumhaft erscheinenden Nachtarbeit*).

xxiii. "But the most beautiful thing about my burrow is its stillness... Of course, that is deceptive. At any moment, it may be shattered and it may all be over" [10], p. 327.

xxiv. "I find myself sensing an atmosphere of great danger" [10], p. 332. Kafka's *The Neighbor *exhibits a similar paranoid structure, which is expressed in the animal-narrator's remark, "my burrow could not tolerate a neighbor" [10], p. 358). In Kafka's *The Neighbor*, the protagonist attributes all sorts of malicious intent to his neighbor, whom he scarcely knows and without the slightest evidence: "I have never got a good look at him yet, for his office key is always in his hand when he passes me... The wretchedly thin walls betray the honorable and capable man but shield the dishonest. My telephone is fixed on the wall that separates me from my neighbor ... What is Harras [his neighbor] doing when I am telephoning? ...- I must assert that Harras does not require a telephone, he uses mine. [no evidence for this] He pushes his sofa against the wall and listens; while I at the other side must fly to the telephone, listen to all the requests of my customers, come to difficult and grave decisions, carry out long calculations, but worst of all, during all this time, involuntarily give Harras valuable information through the wall. Perhaps he doesn't wait even for the end of the conversation, but gets up at the point where the matter has become clear to him, flies through town with his usual haste, and before I have hung up the receiver, is already at his goal working against me." [10], p. 425, my insert.

xxv. The Muirs translate "my whole body" for the original German "my body in all its parts" (...*meines Körper in allen seinen Teilen*)." The relationship, critical to the current argument, between the burrow and anatomical bodily-parts is not reflected in the Muirs' translation.

xxvi. As Murray (2004) observes, Kafka wrote this story during a period of his life in which he had finally found some contentment but was also afraid of loosing it. For this reason, and for reasons perhaps intrinsic to hypnagogic *Doeppelgaenger *and autoscopic experiences, the whole story is pervaded with paranoia with regard to imaginary doubles (e.g., his hypothetical *Waldbruder*, or the unknown animal(s), later in the story, boring its/their way into his burrow to destroy him).

xxvii. The Muir's translation, "both physically as well as mentally" is misleading as the animal states that the labyrinth causes him ***physical ***(not mental pain): "... *auch **körperlich **überwinden*..." This suggests the arduous path of the fetus during birth.

xxviii. Brentano's student Husserl developed the "phenomenological method."

xxix. Husserl's claim of "genetic" levels of meaning in consciousness (starting from an abstract and non-experienceable living present) is not a claim of microgenetic phases in the development of perceptual Gestalt-meaning, which would be experimentally isolable (as in the tatiscopic experiments of microgenesis) in real time (see [49], for review). That is, the genetic phenomenological "reduction" (see below) is strictly a reflective method. It makes abstract slices into conscious experience by ascertaining the prior conditions of possibility, as if able to remove abstract layers as so many layers of an onion and seeing what remains as prior or invariant in the process, (see [3,48] for reviews of phenomenologic method). Because the living present, or now point, remains abstract and inaccessible to experience [48], it is unable to serve as the basis of what Michel Henry and the neo-phenomenologists, following him, claim to be the *immediate *experience of self in so-called pre-reflective or naive self-awareness (see next section).

xxx. Previously, I indicated that Kafka and Husserl employ the same metaphor of descending into an underworld - or in the German romantic tradition, a descending into a mine (*Bergwerk*) - as indicating an exploration of the depths of self [1,68]. Moreover, both Kafka and Husserl use the image of double to describe the reflective relationship to self as a splitting into I as subject and as object (me), i.e., as "gap" see also [2,3,9,48]. The French psychoanalyst, Lacan defines this gap or split between the "I" and "me" *to be *the "unconscious" *itself*. This differs from Lacan's phenomenologic contemporaries, however, who claimed rather that this "split" belongs to the nature of human embodied self precisely as a process of self-transcendence, i.e., an ongoing emergent and dynamic field determined by a "lability" of thresholds (Schwellenlabilität) [53] between conscious and "unconscious" processes (see [9,19,48,53,62]).

xxxi. "Sure enough, this being in itself of the stream of consciousness is entirely its own sphere of Being by virtue of the fact that its future is conversely not 'in itself'" (Husserl [67], p. 208, my translation).

xxxii. As already indicated, Husserl finds a similar paradox when thinking about or reflecting on our own "current" experience, which, by necessity, is retrospective. Every reflecting on our experience itself occurs within the temporal passing of consciousness and is subject to the same "laws" of "time consciousness" as the original reflected on experience. The very act of thinking about or reflecting requires *a splitting of the "I" (Ichspaltung) into an currently thinking or reflecting "I" and a reflected (already past !)"me*." When we are caught up in perceiving or experiencing things, there is a loss of self (*Selbstverlorenheit*), a naiveté about our role in constructing the experience: "Admittedly, the moment I begin to reflect, the naïve perceiving by the self-forgetting I is already past. I am only able to grasp this by reaching back - in the reflecting - into what has 'remained in consciousness' as retention, an immediate memory which *attaches itself backwards *to the original experience" (Husserl, [58], p. 88, my trans and emphases). I am able to reflect on my original naïve self-forgetting which is absorbed in the experiencing only because the I itself has 'split' (*Ichspaltung*) into a reflecting I and the object of its reflection, the naïve I just previously engrossed in experiencing (i.e., the self now as object or "me"). The splitting or objectifying of one's subjectivity as past is passive and occurs automatically. It is not as Zahavi and other adherents of the largely Henryan interpretation of Husserl (see below) propose, that reflection "introduces" the splitting.

xxxiii. As I [48] have recently proposed, the "phenomenological unconscious" (Husserl) is two-pronged: it is both the lowermost stratum of the primal sensory impression (*Urimpression*), or "living present," i.e., the pre-affective syntheses *underlying *the background of any emergent Gestalt (which, in turn, affects consciousness in its emergent contrast saliency over against its background), but it is also the past, the "night" of the unconscious, into which every emergent saliency passes, progressively loosing its affective contours and ability to attract awareness. This self-dividing (*Entzweiung*) of time consciousness (as self-displacing totality), is necessary for the memory of a past self, so that *the two selves*, past and present *are related in terms of the hidden unity *of a Gestalt-circle or revolving door principle. The moment I become narratively entranced with my past self, my present self goes in the background, but to the degree that "I" narrate this past, by virtue of narrating it, "I" transcend it as an already past "me" embedded in the past situation, and thus obtain the provisional narrative *mastery *of a traumatic past in the telling of it [18]. Critically, the unconscious in the Husserlian sense (*as *enabling this self-dividing of its own experiential field) is not the perceptual or experiential background, but underlies it, making it possible [48,67,68].

xxxiv. That is, precisely to the extent that the neophenomenological approach bases itself on Henry's concept of ipseity, it *distances *itself from the phenomenological tradition [71]. It is both "neo-" but also recalls some of the dogmatic pronouncements of a theologically invested scholasticism. Henry interprets his own work as "antithetical" to his phenomelogic predecessors (e.g., Gadamer, Heidegger, Husserl, Merleau-Ponty, Sartre, Scheler) precisely because he extends the "phenomenologic" project *far beyond *what any of his predecessors deem possible, and in a way that contradicts them. For example, he proposes that the human subject, as natural, pre-reflective or naïve bodily consciousness, is the realization of its own essence through the manifestation of immediate "absolute knowledge" of itself (in autoaffection) as "Being in itself." Readers acquainted with the other phenemenologic authors listed above will no doubt see in Henry's proposed pre-reflective self-awareness a rebelliously barbed contradiction to each of their methods and main theses in turn. I discuss this fundamental discontinuity with the "phenomenological tradition" (Gadamer) inherent in Henry's concepts and their neophenomenological appropriation elsewhere [6,71].

xxxv. From the Latin for self, or itself, *ipse*. Henry's use of the term "ipseity" (embraced by his hyper-reflexive neophenomenologic adherents) is idiosyncratic and should not be confused with Ricoeur's definition which does not overlap with Henry's.

xxxvi. What Henry writes about the living body and its double as object representation could just as well be said about the body subject and its hypnagogic hallucination as double, whereby the patient becomes confused (in Type II dreamlike-delusional autoscopy) which body is really his and which belongs to the double. That is, the relationship between the lived-body as subject and its double is "symbolic." Nevertheless, what Henry [73] means here by linguistic "symbolism" differs from my definition of the "symbolic" doubling of the self in Kafka's literary narratives and a general characteristic of hypnagogic hallucinations [2,3,47,48].

xxxvii. The Gestalt-circle occurs according to a "revolving door principle" (*Drehtuerprinzip*): "Each act is perception and movement. However, I am unable to perceive in my perception the movement that made it possible. Conversely, I am unable to access in the movement the perception that guides it. ... Movement and perception stand in a relationship of mutual concealment" (Von Weizsäcker, [53], p. 200, my translation). The relationship is circular in the sense that one is "unable to ever establish where the relationship begins or ends" (von Weizsäcker [77], p. 26; my translation).

xxxviii. This is not merely a philosophic debate, a battle of egos (as suggested by my "neophenomenological" colleagues responses to my criticisms so far), or pedantic, hair-splitting arguments about how to interpret a tradition of texts. What is at stake is how to conceptualize and study the human self and its disorders, and to what extent the phenomenology of the subjective experience of the patient may contribute to neuropsychiatric research. My colleagues' marked defensive intolerance of disagreement (in their published responses up to this point) unfortunately more reflects on their own experience of vulnerabilities in their arguments, and how they elect to cope with them, than on the ability to muster some good will to help advance the field through poised academic debate concerning issues of substance. So far, they have responded to criticism by providing (irrelevant) ad hominem arguments, taking my statements out of context, quietly changing their own position as if it already included whatever I criticized (i.e., presenting moving targets) by posturing, "we meant this all along," etc. I refer the interested reader to the upcoming issues of *Philosophy, Psychiatry and Psychology *devoted to this debate, and our contribution, "The phenomenology wars: self, psychopathology and neuroscience"[71].

xxxix. Phenomenological method (as proposed by Husserl) offers a disciplined sequence of steps: 1) Phenomenological reduction is a "leading back" (from the Latin *re-ducere*) from one's current engagement with the world to examine (reflectively) the "streaming-consciousness" in the here and now; this requires the bracketing of common sense folk-psychological/folk-physical assumptions about how minds and objects behave in the world; 2) Abstracting the essential meaning-structure of an object by bracketing (or suspending) its reference to reality and examining its limits by freely imagining variants which fall within its semantic boundaries ("eidetic, imaginative variation"); 3) Rigorously describing the "results" in a technical language which is as sensitive as possible to fine details of the experiencing while monitoring this language for lapses into reification of our common sense folk-psychology; 4) Integrating the findings into a theoretical framework which is then imparted to a community of investigators for "replication" using the same method. The phenomenological method (which I present here in a simplified manner) has been criticized as being less transparent than it claims (e.g., Gadamer [8]). It is very difficult to explicitly follow a method without implicitly employing the eventual "expertise" that accrues through practice (i.e., the procedural know-how and accompanying "prejudices" (*Vor-urteile*) we acquire over time and apply without awareness) (See Mishara [6]). Similarly, Merleau-Ponty [59] observes that phenomenological reduction (i.e., reflection on our experience) is inevitably mediated by language even when we claim to be describing nonverbal, pre-linguistic, "mute" sources of meaning (see [48]). This problem becomes even more pronounced when one claims access to an "intuition" of life which is pre-reflective, pre-linguistic *and yet captured in reflective, verbal concepts about that experience *(as the neophenomenologic pre-reflective ipseity), thus, recalling a confound identified in the experimental literature as the "verbal overshadowing" effect.

xl. Every reflecting on our experience itself occurs within time and is subject to the same "laws" of "time consciousness" as the original reflected on experience. The very act of thinking about or reflecting requires *a splitting of the "I" (Ichspaltung) into an currently thinking or reflecting "I" and a reflected (already past !)"me*." Husserl writes: "Admittedly, the moment I begin to reflect, the naïve perceiving by the self-forgetting I is already past. I am only able to grasp this by reaching back - in the reflecting - into what has 'remained in consciousness' as retention, an immediate memory which *attaches itself backwards *to the original experience" (Husserl [58], 88, my trans and emphases). The splitting or objectifying of one's subjectivity as past is (contra Zahavi) passive and occurs automatically.

xli. Held criticizes the phenomenologist, Gerd Brand's argument (later adopted by Zahavi and other advocates of the neophenomenological position) that since we have reflective self-awareness, this "*must*" be preceded by pre-reflective self-awareness. Gerd Brand calls this pre-reflective source of reflection, "rudimentary reflection" (*Reflektion im Ansatz*). Held writes: "As these assertions are made from the standpoint of reflection, they either remain empty in terms of any positive content or not to be taken literally at their word" [66], 105 my trans). It is puzzling then, that Zahavi should cite Held as supporter for his concept of pre-reflective self-awareness.

xlii. In sleep paralysis (a symptom of narcolepsy), the patient experiences an inability to move when falling asleep or upon waking. Although conscious of the surroundings, the patient may, at the same time, have nightmarish hallucinatory experiences [3].

xliii. As reported above, individuals isolated for long periods (e.g., mountaineers, explorers, sailors, and castaways) report a variant of the *Doppelgänger *experience, the "feeling of a presence" (FOP) in which another subject is *felt *to be in the self's proximity but not seen. "The phenomenological-psychiatrist, Jaspers [60] noted that it is not possible to classify the feeling FOP (*leibhafte Bewusstheit...*) as a perceptual hallucination or as a delusional belief. Nevertheless, it may harbinger a subsequent "transition" to hallucinations and/or delusions. FOP may serve as a stage in the development of these other psychopathological phenomena and continue to be a component of these experiences. For example, prior to the development of more florid symptoms of active paranoid psychosis, prodromal schizophrenia patients may feel they are 'watched' or 'observed' without anyone nearby [60]. Although the idea - as far as I know - has not received attention in the current literature, the phenomenology and underlying neurobiological mechanisms of FOP may indeed support the view that FOP is a critical component in the development of delusions and hallucinations in schizophrenia. (Mishara [3]).

xliv. Here we have a doubling between the protagonist and the artist but also a second doubling between the artist and Kafka as writer. As noted above, the painter-artist in *the Trial*, Tintoretto, attempts to provide a portrait of the protagonist, Josef K., in parallel to Kafka's own efforts to develop in writing a "portrait" of the same character.

xlv. Interestingly, this relationship of mutual exclusion extends to the remembering of dreams. "Autobiographical" episodic, or narrative memory is what the individual ***explicitly ***remembers about the course of experiences. Each experience is 'tagged' in explicit episodic memory as having occurred at a specific time and place in the person's life (even if this time and place are not always recalled with precision). Husserl calls this contextual tagging its temporal place, "Zeitstelle." The phenomenological psychiatrist Wyss [106], who had trained with both Jaspers and von Weizsaecker, describes dreaming as the "loss of perspective" or a-perspectival. Since dreams are not experienced by an awake, embodied-self, embedded in identifiable space and time, they are not directly accessible to autobiographical memory. It is only when we recall our dreams that they become accessible ***as remembered***, ***as having occurred at this particular place or time in our lives ***; i.e., a spatio-temporal context - presumably mediated by medial temporal lobe structures - is not provided during the original dreaming but only when it is subsequently remembered. I have proposed that traumatic memories which are often just fragments which return in an unbidden, intrusive manner during both awake experience and nightmares are not available to the conscious autobiographical memory system. It is only by narrating them (not just experiencing their intrusive recollection in flashbacks), i.e., by either writing [18] or speaking them that they transfer from an unconscious fear-conditioned memory system to a more conscious and voluntarily available episodic memory system. That is, narrating the trauma is healing and brings a feeling of "mastery," because it transfers "piece by piece" the fear-conditioned traumatic learning from the unconscious to the more voluntarily accessible conscious memory system. The patient experiences closure and less vulnerability to the unbidden flashback episodes [18]. In subsequent publications (in collaboration with Dr. Catharina Bonnemann, Medizinische Hochschule Hannover), we examine how the narrator's embodied "point of view" (and corresponding transform of reference frame) is critical for the narrative's healing power.

xlvi. "Niemand wird hier lesen was ich hier schreibe," says Graccus. The English translation tries to soften this contradiction by incorrectly translating this as, "Nobody will read what I have to say here."[10], p. 230. Kafka employs a device of self-reference built into the narrative to show that the narrator as "I" is always concealed behind the content of his narratives. Similarly, the film director Wim Wenders weaves his signature into the content of the filmic images to demonstrate an irreparable breach between author and audience via the non-transparency of narrative content back to its creator. Such references to the creator built into the filmic image may be found at the end of "Kings of the Road," where all the letters of the movie theater's (!) neon sign *Weise Wand *(literally meaning "blank screen) are burned out except for the first W's of the two words (the initials of Wim Wenders own name). Thus Wenders autographs the end of his film in the way that a painter signs his painting. In Wenders' "State of Things," the film director, Fritz who is trapped with his cast in an abandoned Portugal seacoast hotel without any film to continue production. He complains to his cast that there is so much going on right now in a random sort of way, even as he is speaking to them. They could capture this with a few cameras but they have none. He says, "There is no film in the camera. A lot things are happening simultaneously. If we had more than one camera, but we do not even have one; [and then contradicting all that he has just said by now referring to the film as indeed the precondition for the audience’s experience] All this is fiction." While the reported absence of a camera may be true of the narrative of the film's story which is about the interruption of a film's production due to the absence of film, it is not true of the film which the audience watches. That is to the extent that audience becomes entranced by the filmic narrative, which reports that there is no functioning camera, they must suspend their more immediate experience that they are watching a film clearly captured by a movie camera. Similarly, Magritte points to the paradox of self-reference in the pictorial image in his celebrated picture of a pipe, entitled, "This is not a pipe." That is, narrative, even the pictorial or performance type of narrative previously discussed, establishes a non-transparent relationship between author and audience by creating the illusion of narrative, as the framing of imaginary time within real time whereby the audience experiences a vulnerability to becoming absorbed or embedded in the scenes depicted. We also saw this in the relationship of trance between the medicine man and his audience who must also put himself in a trance to become convincing. However, this non-transparent relationship between narrator and his/her narratives, which encourages artists such as Kafka or Wenders to insert their own initials into the body of the text as a kind of reminder, is not only between artist and audience but also of the artist with his/her own self during the narrative act.

## Competing interests

The author declares that they have no competing interests.
